# ACTIVE involvement in alcohol care: a community case study in coproduction

**DOI:** 10.3389/fpubh.2026.1816664

**Published:** 2026-06-18

**Authors:** Vicky Best, Lucy Bryan, Stan Edmunds, Nancy Kitch, Elizabeth Sastre, Kerry Wells, Michelle McNeice, Lynn Owens, Mandy Smith, Ravi Menghani, Hannah Sharp, Paul Richardson, Janet Garner, Mick McKeown

**Affiliations:** 1 ACTIVE, Liverpool, United Kingdom; 2 Faculty of Health and Life Sciences, University of Liverpool, Liverpool, United Kingdom; 3 Department of Hepatology, Royal Liverpool University Hospital, Liverpool, United Kingdom; 4 CHAMPS Public Health Collaborative, Wallasey, United Kingdom; 5 School of Nursing & Midwifery, University of Lancashire, Preston, United Kingdom

**Keywords:** alcohol care, coproduction, lived experience, nursing leadership, patient and public involvement, peer support, service development

## Abstract

This is a descriptive case study of a public engagement group of people who have used alcohol care services. Involvement of people with lived and living experience is increasingly recognized as essential in health and social care policy. However, in alcohol care services, such involvement remains underdeveloped and sometimes tokenistic. This community case study outlines the establishment, development, and impact of the ACTIVE (Alcohol Care Team InVolvement and Engagement) group—a pioneering public and patient involvement and engagement (PPIE) initiative in alcohol care services across Merseyside and Cheshire (UK). The group was established through collaboration between the Programme for Alcohol Care and Treatment (PROACT, a regional public health network bringing together all alcohol care providers), public health commissioners (Champs Public Health Collaborative^1^), and the Comensus PPIE model at the University of Lancashire. It was designed to create safe spaces for meaningful coproduction and deliberative democratic communication between people with lived experience, practitioners, and commissioners. ACTIVE has contributed to service design and evaluation. Additional impacts include supporting digital innovation, strengthening regional networks, influencing innovation, and enhancing workforce education. Members reported enhanced confidence, sense of value, and shared ownership of service development. The approach illustrates the replicable potential of sustained, well-resourced involvement of people with lived experience in alcohol care, having implications for policy, research, innovation, and service delivery nationally and internationally. The case study has been collectively authored utilizing notes of meetings and drawing upon contributions within a series of reflective meetings and exchanges of drafts.

## Introduction

In this paper we describe and reflect upon a novel participatory public engagement process for improving and developing alcohol care. We contend this is congruent with wider aims to democratize public administration or health systems in the interest of collective good and, indeed, the importance of democracy generally for supporting public health and mitigating risks (1–3). We reflect on our work to establish and develop the ACTIVE group and discuss this in relation to available theories of public engagement and participatory democracy.

There is a commitment across health and social care to enhance involvement of people who use services and family carers in decision-making and coproduction (4, 5). Such practices have improved the quality, relevance, and responsiveness of services. However, in alcohol services, involvement remains less developed (6, 7, 53, 55). A lack of meaningful engagement can be compounded by a judgemental stance within services toward people with alcohol problems, arguably limiting effectiveness of care and support (8, 9, 52). Service users report they lack control and agency in navigating services (9) but are united with practitioners and commissioners in defending the need for good services (10).

This makes a persuasive case for establishing systems for supporting and attending to patients' voice within alcohol services. The sensitive and stigmatized nature of problematic alcohol use compounds organizational challenges (9). Nonetheless, peer support traditions demonstrate the capacity of people with lived experience of problematic alcohol use to offer meaningful engagement and leadership.

In the spirit of coproduction this article has been written using a democratic approach, bringing in contributions from people with lived experience, commissioners, practitioners and group facilitators. We organized several reflective meetings to establish interest in writing a piece for publication, to support the writing of the paper, and decide where would be the best outlet for our work. Our writing process involved taking notes of discussions, drafting sections of text and taking group feedback to refine and ensure key messages or experiences were included. Nine members in total have been involved in reflective meetings with seven participating in the full writing process. We reflect a collective voice in presenting our work, relying on “we”, “our” and “us” to address readers. We make an exception at junctures where we distinguish professional and lived experience voices, particularly at times to privilege the importance of an unadulterated service user voice.

## Context

Establishing involvement practices in this field is complicated by prevailing sensitivities (7). Previous inquiry has revealed involvement practices related to service development can be experienced as overly prescriptive, rather than more meaningful, continuous approaches defined by coproduction principles (6). There are, however, some interesting involvement practices in the context of research into alcohol care provision and related experiences, including researching peer support within services (11, 12). Recruitment of people with relevant lived or living experience into patient or public involvement and engagement, for example, guiding a research project, can be a requirement of commissioners or research funders. Institutional acronyms such as PPI or PPIE can be confusing for people asked to take part (13). Having people with relevant lived experience as co-researchers into alcohol services is increasingly evident in the literature and helps deepen mutual respect between peer and established researchers and raises the possibilities for capacity building such that peers may eventually lead on identifying and addressing their own research questions (14). It is our experience that being asked to be involved at the earliest possible opportunity within a research process, and continuously throughout, is most effective.

### Formation of the ACTIVE group

Data published by Public Health England (15) demonstrated a 7.4-fold difference in alcohol-specific hospital admissions across England, with the highest rates concentrated in areas of social and economic deprivation, and significant and unwanted variations in service provision, clinical practice, and outcomes. Cheshire and Merseyside consistently rank high on national indices of deprivation, reflecting a substantial regional burden of alcohol-related harms.

**Figure 1:**
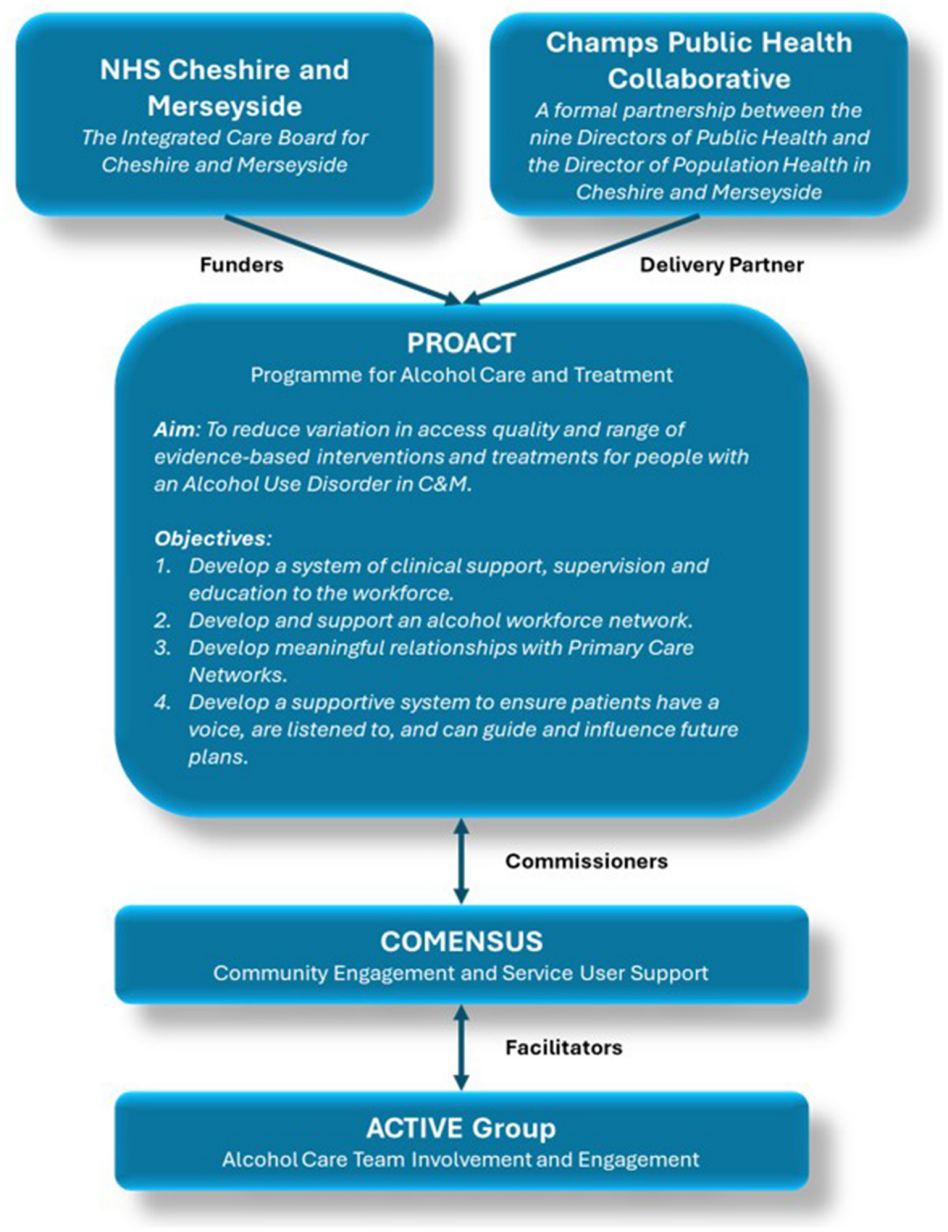
Organizational relationships.

In response to this, in 2020, the relevant public health network, Champs (Cheshire and Merseyside Public Health Collaborative; see text footnote 1) established the PROACT collaborative (Programme for Alcohol Care and Treatment in Cheshire and Merseyside) which aims to reduce variation in access and quality of alcohol care in Cheshire and Merseyside. This initiated a programme aimed at developing a unified system of care delivery. The goal was to ensure individuals presenting with alcohol use disorders received consistent, safe, effective, and compassionate care across all participating services. Central to this vision was recognition that service users' lived experiences were essential to identifying priorities, shaping service design, and driving meaningful change. The ACTIVE group was established as a strategic partnership between the PROACT team consisting of local senior alcohol specialist nurses and Champs public health commissioners, and the Comensus (Community Engagement and Service User Support^2^) patient and public involvement and engagement (PPIE) initiative at the University of Lancashire [see Figure 1]. Comensus is a long-established public engagement programme that supports education and research at the university, involving a substantial and diverse network of participating individuals and affiliated community groups. Developed as a participatory action research project, Comensus has maintained and refined democratic approaches for involvement, tapped into people's motivations for change, and researched these in practice (16, 17).

The PROACT team led on the development of a comprehensive business case, which secured funding from NHS Cheshire and Merseyside (the Cheshire and Merseyside Integrated Care Board). To ensure long-term sustainability and accountability, a Memorandum of Understanding was formalized between partners (see Supplementary materials). This outlined shared objectives, governance arrangements, and expectations regarding the group's role in influencing service development beyond the lifespan of individual projects or short-term funding.

### Group ethos

A key focus of the first wave of engagement between PROACT and ACTIVE sought group members' input into addressing pathways of care for individuals with co-existing mental health and alcohol care needs. This supported development of a new service aimed at preventing people falling through gaps between services and improving continuity of care. Consequently, the ExAMH (Early Identification of Co-occurring Mental Health and Alcohol Issues) pathway was piloted. ACTIVE played a key role, informing assessment tools and outcome measures, advising on staff practices emphasizing relational care, and involvement in external evaluation of the new pathway.

Over the last 5 years ACTIVE have gone on to be involved in a series of novel initiatives. They are now considered a standing PPIE resource to alcohol care services across the region and support research projects in the field led by colleagues at Liverpool University. ACTIVE is one of only a few examples of a well organized and enduring PPIE initiative focused solely on alcohol care within a UK region. Moreover, it has several other notable features. The group adhere to specific core values and democratic practices: there is a flattened hierarchy, warm mutual respect, support across difference, mutual emotional support, sophisticated reciprocity, and deliberative communication. The latter aimed at ensuring constructive ideas emerge from discussions, with plural solutions not neglected in a simplistic desire for consensus. Differences of opinion are encouraged and explored in reasoned debates. In accomplishing this democracy, ACTIVE members draw upon previous experiences, such as within various peer support approaches, offering attentive listening, challenging, and democratized communication. ACTIVE have also taken care to meet in comfortable community settings conducive to support and communication, such as the Liferooms hosted by Mersey Care NHS Foundation Trust. This has also allowed participants to gain access to wider social support and activities.

We recognize a need to distinguish between terms such as codesign and coproduction albeit these are often used inter-changeably in practice (18). ACTIVE members attend to two distinct ways their work is generated. Operating in their own protected spaces, members feel able to identify matters worthy of attention and bring these to the attention of professional colleagues for conjoint consideration. Alternately, the professionals identify issues that are brought to the joint meetings for ACTIVE members consideration. We consider our group to be involved in coproduction as we work in cooperative partnership with professionals to define issues and solve problems, producing a variety of outputs that help to shape or contribute to improvements in services and see the cycle through to involvement in evaluation. Some of our work is perhaps better described as co-design, for instance where we have contributed meaningfully to the design and creation of artifacts such as leaflets or digital applications, often guiding the inputs of professional designers or technologists but the extant need or problem has been pre-defined within services.

## Key programmatic elements

### Recruitment and membership

Recruitment to the ACTIVE group followed established PPIE principles to ensure representation, inclusivity, and diversity of experience. Members were initially recruited through the Comensus network, local alcohol services, and community partners. Each member is provided with an induction outlining the project aims, governance structures, and expectations.

### Participants include:

- Individuals with lived experience of alcohol use disorders or recovery journeys.
- Family members and carers, providing perspectives on support needs.
- Facilitators from Comensus

These core ACTIVE participants also meet regularly with:

- Healthcare professionals, including specialist nurses, clinicians, and alcohol liaison staff.
- Service commissioners and public health leads.

Group membership is regularly refreshed, for instance if members decide to leave for personal reasons or changed circumstances. At any one time we have between eight and 10 members. Seventeen different individuals have been involved in total since the start of the group. These comprise 5 men and 12 women, ages range from 36 to 64, with the majority from lower socio-economic groups, a minority are in paid employment, only two members have been from an ethnically minoritised group, many have survived domestic violence. All have experience of accessing a wide range of alcohol care from Emergency Department, through non statutory organizations to residential care, two have survived liver transplants. All members are currently in recovery and are typically abstinent, but we remain vigilant to the possibility of relapse and the need for an empathic supportive non-judgemental response. We also have direct access to a wide range of support should any member lapse into drinking; to date we have not had recourse to use this. New members are recruited from the aforementioned networks and also by recommendation of established members.

### Roles and function

The ACTIVE group functions as an advisory and coproduction forum, providing insight and guidance at all stages of service development. Members have contributed to:

- Identifying priorities for improvement in alcohol care pathways.
- Reviewing patient feedback and experience data.
- Coproducing patient information materials and staff training resources.
- Advising on compassionate communication and trauma-informed approaches.
- Supporting dissemination and evaluation of project outcomes.

Regular meetings of the ACTIVE group (monthly) are facilitated by Comensus staff, ensuring equitable participation and psychological safety within discussions, with separate quarterly meetings facilitated by PROACT nursing staff to feed ACTIVE deliberations into regional strategic and operational thinking and for PROACT members to directly seek the views of ACTIVE on emerging ideas or developments. Group members also informally keep in touch using WhatsApp. Meeting agendas and topics for discussion are democratically agreed between ACTIVE members and professionals. These are assembled and circulated in advance of meetings and notes are taken. The conduct of meetings is relatively loose, informal and deliberative.

#### Principles and structure

The work of ACTIVE is underpinned by four principles (see Supplementary materials for more information): (1) Flattened hierarchy–shared decision-making and flexible, light-touch facilitation ensuring equal voice among members. (2) Safe peer spaces–peer-only sessions foster confidence and reflection before wider engagement with professionals, supporting autonomous organization and trauma-informed participation. (3) Deliberative democratic communication–with reflective listening and open dialogue to integrate diverse experiences. An appreciative stance toward each other is maintained. These deliberations are acknowledged to have a powerful affective dimension, and the group demonstrate capacity to hold strong emotions and respond supportively. (4) Community-based engagement–sessions are held in accessible venues, reducing intimidation and promoting inclusivity.

From the outset, we have taken steps to organize protected, safe spaces for the ACTIVE group to share their experiences and ideas freely, support extended peer-led discussion on agendas set by the PROACT team and independent generation of topics. Time is given to all to voice their perspectives and offer suggestions before we reach consensus, or agree that different perspectives are valid; for instance leading to legitimately plural demands on services.

The character of meetings are typically warm and appreciative, with plenty of humor and creativity demonstrated. This is not to say there are no challenges or conflicts. Our foundational values are useful for pre-empting conflict and facilitators and group members themselves are variously skilled in calming down tense situations or mediating disputes. The reality is that we are relaxed about differences of opinion and members are quite skilled at communicating respectfully, so we have not had much in the way of serious conflict. A degree of turbulence, for instance in advocating for change with professionals in a context of straightened resources to enable extensive change is acknowledged and we concur with the idea that this is healthy (19).

### Outcomes

ACTIVE has achieved multiple outcomes, including:

- Coproduced pathway for co-occurring alcohol and mental health disorders.
- Coproduced suite of Competency Frameworks for Alcohol Specialist Nurses, Care Workers, and Specialist Nurses providing contemporaneous care to our patient group.
- Coproduced guidelines for introducing and supporting: Multidisciplinary Team working, Volunteers working in services, Screening pathways to detect alcohol exposed pregnancies.
- Informed digital innovation (Champs commissioned ‘Lower My Drinking' mobile phone application).
- Coproduced educational materials for the PROACT education programme.
- Strengthened regional approaches to care via contributions within the PROACT network.
- Reduced workforce stigma through storytelling and training that humanized alcohol-related care.

These achievements notably include establishment and evaluation of an entirely new service (ExAMH) and numerous refinements to practice and practitioner education across the region. The latter includes helping to produce a new workbook for student nurses (see Supplementary materials), supporting their learning within alcohol services and producing a series of short films promoting the value of services from a lived experience perspective. Participants reported that ACTIVE “humanized” services traditionally experienced as overly clinical. Service users became valued partners rather than passive consultees. Our approach has fostered:

- Compassionate communication.
- Mutual respect between lived experience participants and professionals.
- Increased confidence, empowerment, and skills among ACTIVE members.

Table 1 shows a more detailed representation of ACTIVE activities and outcomes; Supplementary materials provide more information.

**Table 1:** Overview of ACTIVE group activities and outputs^a^.

Domain	Example activities	Outcomes/impact
Service design	Co-produced dual diagnosis pathway	Improved integration of alcohol and mental health care
Competency framework	Co-produced a skills and competency framework for nurses	Improved and standardized skills within and across ACTs
Education	Student nurse workbook	Enhanced empathy and reflective learning
Digital innovation	Consultation on “Lower My Drinking“ app	Lived experience-informed digital support
Network strengthening	Embedded in PROACT collaborative	Ongoing cross-sector collaboration
Workforce culture	Storytelling and training sessions	Reduced stigma and improved communication
Resource creation	Developing scripts and short films based on ACTIVE's lived experience and knowledge of participation.	Raise awareness of the issues surrounding this group of service users and combat stigma. Specifically targeting post-graduate researchers but with broader potential usage.

^a^Where available additional information about these outputs has been provided as Supplementary material.

#### Participant experiences within ACTIVE

Having lived through similar experiences, there is a common bond between the members of ACTIVE who identify with lived experience. Participants speak movingly about how empathy and understanding enables them to get the best out of each other and achieve optimum results within the group. Humility has been singled out as one of the cornerstones of group relationships. As an aside, health professionals and commissioners who meet with the group report finding the life stories, particularly biographies of recovery from very challenging circumstances, humbling to hear and inspirational for future practice.

Ideals of mutual compassion and empathy are also expressed as key to the functioning of the group, and this can be contrasted with negative experiences of encounters with certain health professionals in the past. There is a commitment, often vocalized, that every one of the members deserve to be treated with compassion. Participants are not shy of talking about affective components of group relations, for instance talking about leading with love and sensitivity as we don't always know where people are on their healing journey, how they might be feeling in the present, or how prepared they might be for discussing sensitive issues.

Although we are all still very much working on ourselves and trying to be better than we were yesterday, we accept that each one of us plays an important part within the group. We listen without judgement and there is no competitiveness, which we feel sets a different standard within our group. We accept each other without judgement, as and for whom we are, which means any sense of shame is banished. We respond to one another with warmth and positivity. We believe this sets us aside from other groups, where we have sometimes been interrupted and responded to without much care or compassion. Our group most prioritizes both care and compassion, which is key to achieving the best results from service users: in a participatory group like ours but also within services. These are certainly some of the challenges we have faced within differently constituted meetings. We are more than just a number, a statistic, and we feel we should be treated with recognition, care and respect.

We are all extremely committed to the group. We are committed to making a difference and getting our message across. The presence of ongoing battles within our lives is what drives us to keep going and pushing forward. To challenge stigma and find new ways to gain support.

### Discussion: practical implications and lessons learnt

#### Challenges

Whilst mostly everything that has been planned has gone well, there have been some frustrations and challenges. Full coproduction where the group have taken a lead on identifying and seeing through changes has been most appreciated. The group have also responded positively to requests to contribute to initiatives that were already part designed or in progress but have argued earlier involvement would have been preferable and more beneficial. In one example, consultative relationships with a research team have been put on a firmer footing and there will be more timely involvement going forward.

In one PROACT meeting, despite pre-emptive briefings of professional staff not to use stigmatizing or moralizing language, one member of staff from a voluntary sector organization spoke in a pejorative way about women who drink during pregnancy. This caused upset for lived experience participants necessitating immediate support and an intervention with the speaker. This sort of thing shows that best laid plans can always be derailed and the importance of agile support.

#### Democratized participation

There are many excellent examples of coproduction in the health care context; our ally organization Comensus being one (20, 21). There has also been a long tradition of formal and informal peer support within alcohol care services; systematically organized opportunities for stakeholder involvement are less fully developed and inconsistently organized (6). To the best of our knowledge, ACTIVE is distinctive in establishing a fully participatory and enduring public engagement initiative relating to alcohol care, demonstrating the value of sustained, well-funded, and democratically structured PPIE. ACTIVE aligns with theories of ‘caring democracy' (22) and deliberative participation (23, 24). As such, experiences in ACTIVE connect with various available theories and literature concerning public engagement and participatory democracy.

This constituency of patients and their families are seldom heard in services and other contexts, often being stigmatized, with their voice ignored or silenced. This poses various challenges for services to live up to ideals of consulting with service users and families or engaging in more sophisticated coproduction. For us, the original impetus for addressing these issues was a noted deficit in provision of care for individuals with co-occurring mental health and alcohol use problems. This inspired us taking up the challenge of forming and supporting the ACTIVE group to first focus on a new service for this dual-diagnosis group and then expand its remit to all alcohol services within the region. One of the inspirations to take part was from nursing staff in services hearing the life stories of patients and listening to their wishes for help and support that were not always within the scope of services to deliver. It was acknowledged that a more formal, structured and supported approach to patient participation and coproduction was needed and this should be organized autonomously but in close liaison with services.

There is importance in getting the process of public engagement correct. Conceptual analyses reflect idealized democratic forms that conceive of a collective, public commons, exemplified by democratized and deeply relational communication between civic actors (3). Latterly, health care organizations have embraced the concept and practices of coproduction, democratized collaborations between stakeholders operating on an equal footing, as a way forward for service developments and policy formation. The practice of coproduction, in our view, best describes ACTIVE's relations with organized services and commissioners. The concept of coproduction has a lengthy provenance in community organizing initiatives and has been successfully enacted in the places and spaces of health care delivery, including some seemingly unpropitious environments (25, 26). If democratization is the touchstone of effective coproduction, then we have recourse to various theorizing and experience which points to ways that democracy in practice can be conceived and optimized (27).

Hendriks and Dzur (28) show how coproduction can be misrepresented as an unhelpful binary between the two extremes of democratic grassroots capability to solve complex problems or the unfair shifting of responsibilities onto citizens within neoliberal states. The reality is more complicated and is exemplified, across a variety of civic action fora and self-help groups, by a notion of disruptive policy and practice work. A corollary is the transformation of occupational identity on the part of institutional supporters of coproduction into a form of democratic professionalism (29). We identify with such ideas in our involvement work and see evidence of this democratized professional identity in the colleagues we connect with across the PROACT network.

Philosophers of democracy such as Jurgen Habermas have pointed out various crises and deficits of democracy in society. Such theories have in turn been criticized by a range of political, feminist and disability scholars for various exclusions and a perceived focus on an idealized, polite civility grounded in a conceptualization of rationality neglectful of constraints on power and agency in the public realm (30–33). That said, most critics consider participatory democracy to be worth persevering with and there is a continuing advocacy for refinements of deliberative approaches (34).

Attention to notions of democratic deficit is redolent in a micro sense of the silencing of voice that ACTIVE group members have felt previously. Habermas advocates deliberative, dialogic communication as an ideal form of how to do democracy, where the quality of communication is an essential ingredient of social change, and these ideas have been utilized to frame participatory involvement and engagement initiatives (35), including our own. Habermasian deliberation relies on certain setting conditions, which may be relatively prominent or absent in organized involvement fora. These require the facilitation of fully democratized deliberative process to be grounded in: efforts to equalize power relations between members and facilitators; respect for each other across difference; commitment to have an open mind to persuasion; civil discourse techniques such as listening and not talking over each other. We have endeavored to follow such principles in our meetings, and the reported feelings of respect, care and recognition reflect this.

#### The material, relational and storied spaces of participation

Issues of democracy, participation and power are also influenced by the situated character of the places and spaces wherein participation occurs, and whether these are invited, institutionally implemented opportunities, or have been developed organically by citizens themselves (36, 37). New geographies have considered these places and spaces of democratic engagement, including in the mental health context and highlighting distinctions between material-physical space and relational space (38–40). Our choice of community venues for meetings has involved picking comfortable and well equipped built environments and paying close attention to our interpersonal relations when meeting–nicely reflecting these material and relational notions of place and space.

Other emerging analyses within healthcare environments point to the, often profound, impacts of taken for granted or “mundane” materialities in the lives of service users. This highlights “how [such] materialities help people to balance the expression of their vulnerability with a need to retain their dignity, a practice referred to as “holding one's own” (14, 41), which appears to us to have substantial explanatory appeal in the context of taking care to organize the settings within which our participation is hosted and the compassion and care we extend to each other. We have paid particular attention to how we support each other and build confidence to “hold our own” in more equal conversations across power gradients, with practitioners, commissioners and academics for instance. This has extended to feeding back to meeting organizers about the mundane materialities of seating arrangements at events, or constructively criticizing the language of certain professional contributors.

The character of participatory deliberations and dialogue is typically flavored with storied contributions foregrounding biographical elements and emphasizing lived experiences to inform services, research or policy (42, 43). In this way, the discourse of public engagement groups is often framed by the telling of personal stories, for instance to evidence a point being made or underline its importance (42, 43) and our group is no exception. This is perhaps unsurprising, as we are all storied and storying beings and it would be near impossible to offer views on service quality or change without emphasizing our stories of experiencing care.

#### Foregrounding emotions

Perhaps because of the intimate entanglement of the personal and the procedural within participatory fora, the talk between members also often has a strong affective quality; where emotions are never too far from the surface (44–46). Critical commentators have pointed out how many of the archetypal accounts of deliberative democracy, seem insufficient for guiding or understanding participatory engagement in health, disability or mental health contexts. This is because these theories are alleged to under-value emotion-laden expression because of a too rigid focus on rationality and thus operate to marginalize certain groups. Though it is also the case that public expression of emotion can serve to exclude some people (31, 47). From such perspectives, the expectation for polite civility within deliberative models fails to appreciate the powerful, activist-oriented emotions that can motivate involvement in the first place. Yet, counter critics, such as Neblo (56) bemoan an apparent misrepresentation of classic deliberative theory as depicting emotion in opposition to reason, conversely arguing the original theorizing was always comfortable with emotion as a component of democratic dialogue: rather the exercise of arbitrary or dominating power is the real enemy of rationality. In our experience, we have managed to navigate this potential pitfall, being able to offer highly reasoned, and reasonable, accounts to inform service change whilst also holding powerful emotions within our deliberations.

Our commitment is to be supportive of each other and endeavor to resolve tensions compassionately and empathically or offer support to group members who find certain subject matter upsetting or triggering. In the latter regard, the group facilitators have substantial experience in supporting people through emotional crisis and most of the group members are used to both being supported and offering support in other contexts, such as twelve steps or counseling processes. As such, discussions that enter sensitive territory can be punctuated by mutual disclosures of previous traumas, tears and visible emotions, and various pauses, time-outs and transactions of concern and support. All meetings start with checking in with each other around how we are feeling and debriefing afterwards. Members are aware additional supportive resources beyond the group are on hand if needed, though to date we have not had to utilize these. The ACTIVE group undoubtedly have benefited from the involvement of Comensus colleagues, who bring more than 20 years of experience in supporting PPIE and creating the conditions under which this can thrive.

For example, in a recent meeting where we were discussing end of life care in liver disease one member disclosed her brother had just died. The discussion was paused while everyone offered condolences and hugs and checked she was alright. This member reassured us she was happy to continue being involved in this discussion as a way of honoring her brother's memory. She also made it clear that she had plenty of good support from her family and her sponsor and did not want more from us. She went on to make positive contributions to the dialogue and we all checked she was fine before the meeting concluded, and ensured she had a named contact should she need support later.

Commentators on disability rights and user-led social movements have noted how certain forms of democratized communication can better support debate and discussion of movement goals, actions and issues. For instance, Tronto's (57) call for a more caring democracy, echoes Barnes' (24) focus on the emotional side of group interactions, kindnesses and warmth that participants can express that are helpful in moving discussions forward; in processes of care-full deliberation. Interestingly, previous research and experiences within our group have shown that people who use services value kindness above most factors when considering their relationships with practitioners (8). Latterly, such ideas have been taken forward to argue for democratic fora that simultaneously take care over deliberations and focus discussion on care (48). Hodge (54) has remarked how efforts to organize involvement practices can fail to reach ideal characteristics of deliberative democracy. Our experience has been more positive, and we were always determined to adopt empathic compassionate relations with each other and are unashamed of speaking about this in terms of love and care. This did not necessarily begin with an appreciation of previous theory, rather we have found that these theories neatly fit our attitudes which were born out of experience.

#### Peer to peer participation

Progressive conceptualisations of civic society recognize that citizens support each other beyond formal services and ask questions about services amongst themselves, regardless of and independent to any organized participation. Such peer-to-peer support and cooperation can, however, become a resource to institutionally organized public participation (49). Moreover, PPIE initiatives can in turn favor peer support as an approach for delivering services (50). We have addressed both. Our group deliberations exemplify peer-to-peer support, and we have argued for more paid peer-worker roles in services, or for facilitating similar involvement initiatives. We worked with the group facilitators, who are based in a university, to conduct our own literature review on peer worker employment within alcohol care. It is our view, that many of the practices that best support an effective participatory democracy are also apparent in our experiences of peer supported services, such as 12 step programmes and other approaches.

### Future directions

Beyond continuing with core PPIE activities, some future goals have been decided by participants. These include a community play and film festival to challenge stigma, securing diverse funding streams, replicating the model nationally, and expanding involvement in research and policy development.

## Limitations

The demographics of our group show that ethnic minorities are under-represented, and this is something we are attempting to address through proactive recruitment. Otherwise, there is reasonable diversity of participants, and we are pleased that working class individuals with heavy use of services are well represented, this contrasts with a tendency for some citizen engagement initiatives to over-represent middle-class participants (51).

The members of ACTIVE are always enthusiastic and willing to take on more involvement work. This is not always possible given the budget available and a desire not to exploit people's enthusiasm in relation to available remuneration. Similarly, the ways that NHS financial decisions are made in relation to end of year calculations and increasing insecurity of ICB funding has meant monies for the continuation of ACTIVE's work have recently been considered on an annual basis. This can be stressful for all concerned, though happily our local commissioners are passionate about the value of this work and have made strenuous efforts to ensure continuity.

On a more conceptual basis, whilst we feel that ACTIVE demonstrates the sort of democratic characteristics we have referred to in this paper and committed to in the course of writing it, this reflective piece is not the product of rigorous research methodology. As such, this case study is largely descriptive and anecdotal. A future research evaluation would be beneficial.

## Conclusion

The ACTIVE group exemplifies meaningful coproduction, where people with lived experience are genuine partners in shaping humane, relational, and effective alcohol care. The model is transferable, evidence-informed, and bridges gaps between research, service development, and practice. The relative lack of organized approaches to including people who use alcohol services and their families in public engagement might arguably reflect a view of such service users as chaotic and challenging whilst directly involved in services, or relatively reluctant to engage beyond cessation of care and treatment. Certain treatment modalities, such as twelve steps programmes, are, however, redolent with peer support and democratized communications, arguably laying strong foundations for participation within PPIE processes. Whilst setting up our initiative, we were pleased to find that most people invited to participate welcomed the opportunity, and proved adept at capitalizing upon the invitation.

The value of supporting authentic user voices directly into services and opportunities to participate in genuine and meaningful coproduction of care provision is priceless. ACTIVE members are proud that, together with our professional allies, we have helped achieve a number of substantial and valuable changes. Without being too dramatic about it, ACTIVE members believe these supportive contributions are saving lives, helping to rescue people from the misery and deleterious impacts of alcohol problems, and, at the very least, improving engagement with services because of a positive impact on the way care is delivered, with a renewed emphasis on more humane, loving, relational and supportive processes. For ACTIVE, it is not always WHAT practitioners do, or which technologies or tools they have at their disposal, it is HOW these are applied and what values and attitudes are expressed.

## Author's note

This is a community case study as described by the authors. We are a combination of experts by experience, academic facilitators, alcohol services practitioners and public heath commissioners writing together in a spirit of coproduction that mirrors the work of ACTIVE. We wish to acknowledge and thank the contributions of various members of ACTIVE not named as co-authors.

## Data Availability

The original contributions presented in the study are included in the article/supplementary material, further inquiries can be directed to the corresponding author.

## Footnotes

2 Community Engagement and Service User Support, Comensus PPIE at University of Lancashire.

## References

[1] Baillergeau E Veltkamp G Bröer C Helleve A Kulis E Lien N . Democratising participatory health promotion: power and knowledge involved in engaging European adolescents in childhood obesity prevention. Health, Risk and Society. (2024) 26:201–21. doi: 10.1080/13698575.2024.2338120

[2] Lee S. Does democracy matter for public health? Int J Social Determin Health Health Serv. (2023) 53:15–29. doi: 10.1177/00207314221126110 36113057

[3] Pera M Bussu S. Towards democratisation of public administration. Int Commons. (2024) 18:164–76. doi: 10.5334/ijc.1385

[4] Ocloo J Garfield S Franklin BD Dawson S. Exploring the theory, barriers and enablers for patient and public involvement across health, social care and patient safety: a systematic review of reviews. Health Res Policy Syst. (2021) 19:1–21. doi: 10.1186/s12961-020-00644-3 33472647 PMC7816359

[5] Raffay J Bryant D Fisher P McKeown M Mills C Thornton T. Co-production: Towards Equality in Mental Healthcare. Monmouth: PCCS Books (2022).

[6] Alderson H Kaner E O'Donnell A Bate A. A qualitative exploration of stakeholder involvement in decision-making for alcohol treatment and prevention services. Int J Environ Res Public Health. (2022) 19:2148. doi: 10.3390/ijerph19042148 35206344 PMC8871873

[7] Madden M Morris S Ogden M Lewis D Stewart D McCambridge J. Producing co-production: reflections on the development of a complex intervention. Health Expect. (2020) 23:659–69. doi: 10.1111/hex.13046 32233053 PMC7321726

[8] Coste S Gimenez L Comes A Abdelnour X Dupouy J Escourrou E. Discussing alcohol use with the GP: a qualitative study. BJGP Open. (2020) 4 bjgpopen20X101029. doi: 10.3399/bjgpopen20X101029 32345694 PMC7330215

[9] Gilburt H Drummond C Sinclair J. Navigating the alcohol treatment pathway: a qualitative study from the service users' perspective. Alcohol Alcoholism. (2015) 50:444–50. doi: 10.1093/alcalc/agv027 25825267 PMC4474003

[10] Roberts E Hillyard M Hotopf M Parkin S Drummond C. Access to specialist community alcohol treatment in England, and the relationship with alcohol-related hospital admissions: qualitative study of service users, service providers and service commissioners. BJPsych Open. (2020) 6:e94. doi: 10.1192/bjo.2020.80 32838834 PMC7488322

[11] Eddie D Hoffman L Vilsaint C Abry A Bergman B Hoeppner B . Lived experience in new models of care for substance use disorder: a systematic review of peer recovery support services and recovery coaching. Front Psychol. (2019) 10:1052. doi: 10.3389/fpsyg.2019.01052 31263434 PMC6585590

[12] Francia L Berg A Lam T Morgan K Nielsen S. “The peer workers, they get it”–how lived experience expertise strengthens therapeutic alliances and alcohol and other drug treatment-seeking in the hospital setting. Addict Res Theor. (2023) 31:106–13. doi: 10.1080/16066359.2022.2124245

[13] Foster R Carver H Wallace J Dunedin A Burridge S Foley P . “PPI? that sounds like payment protection insurance”: reflections and learning from a substance use and homelessness study experts by experience group. Res Involv Engag. (2021) 7:1–10. doi: 10.1186/s40900-021-00324-8 34801090 PMC8605889

[14] Berg A Francia L Lam T Morgan K Lubman DI Nielsen S. Enriching qualitative alcohol and other drug research by engaging lived experience peer researchers in a dual-interview approach: a case study. Drug Alcohol Rev. (2024) 43:1040–4. doi: 10.1111/dar.13724 37503834

[15] Public Health England. Health profile for England: 2017. London: PHE (2017). Available online at: https://www.gov.uk/government/publications/health-profile-for-england (Accessed December 16, 2025)

[16] Garner JM Holmes S McClenaghan S Mallen E Melling A Tayya R. Is anybody listening? Using participatory methods to co-create an impact measure for nurse education. J Participat Res Methods (JPRM). (2025) 6:115–42. doi: 10.35844/001c.12830125812574

[17] McKeown M Dix J Jones F Malihi-Shoja L Carter B Harrison N. Service user involvement in mental health practitioner education: movement politics and transformative change. Nurse Educ Today (Special Issue). (2014) 34:1175–8. doi: 10.1016/j.nedt.2014.03.016 24815179

[18] Vargas C Whelan J Brimblecombe J Allender S. Co-creation, co-design, co-production for public health–a perspective on definitions and distinctions. Public Health Res Prac. (2022) 32:e3222211. doi: 10.17061/phrp3222211 35702744

[19] Worsely J Corcoran R McKeown M Wilson T Holt K. A qualitative evaluation of co-production of research: “If you do it properly, you will get turbulence”. Health Expec. (2021) 25:2034–42. doi: 10.1111/hex.13261 33949751 PMC9615072

[20] Downe S Mckeown M Johnson E Comensus Community Involvement Team Comensus Advisory Group Koloczek L . The UCLan community engagement and service user support (Comensus) project: valuing authenticity making space for emergence. Health Expec. (2007) 10:392–406. doi: 10.1111/j.1369-7625.2007.00463.x 17986075 PMC5060412

[21] McKeown M Malihi-Shoja L Downe S The The Comensus Writing Collective. Service user and carer involvement in education for health and social care. Hoboken, NJ: Wiley-Blackwell, Oxford. (2010).

[22] Tronto J. Caring democracy: how should concepts travel? In: Urban P, Ward L (eds) *Care Ethics, Democratic Citizenship and the State*. London: Palgrave Macmillan. (2020) 181–97. doi: 10.1007/978-3-030-41437-5_9

[23] Hodge SM. User involvement in the construction of a mental health charter: an exercise in communicative rationality?. Health Expec. (2009) 12:251–61. doi: 10.1111/j.1369-7625.2009.00561.x 19754689 PMC5060490

[24] Barnes M. Care in Everyday Life–An Ethic of Care in Practice. Bristol: Policy Press. (2012). doi: 10.46692/9781847428240

[25] Dzur A. Democracy Inside: Participatory Innovation in Unlikely Places. Oxford: Oxford University Press. (2019). doi: 10.1093/oso/9780190658663.001.0001

[26] Ercan S Dzur A. Democracy in unlikely places. Democratic Theory 3. (2016) 94–113. doi: 10.3167/dt.2016.030206

[27] McKeown M Dzur A Fisher P. Co-producing democratic relationships. In J. Raffay, D. Bryant, P. Fisher, M. McKeown, C. Mills and T. Thornton (Eds) *Co-production: Towards Equality in Mental Healthcare*. Monmouth: PCCS Books. (2022) 190–201.

[28] Hendriks CM Dzur AW. Citizens' governance spaces: democratic action through disruptive collective problem-solving. Politic Stud. (2022) 70:680–700. doi: 10.1177/0032321720980902

[29] Dzur A. Democratic Professionalism. Citizen Participation and the Reconstruction of Professional Ethics, Identity and Practice. University Park, PA: The Pennsylvania State University Press (2008).

[30] Dryzek JS. Deliberative democracy and beyond: liberals, critics, contestations. Oxford: Oxford University Press. (2000).

[31] Young IM. Impartiality and the civic public: some implications of feminist critiques of moral and political theory. Praxis Int. (1985) 5:381–401.

[32] Young IM. Inclusion and democracy. Oxford: Oxford University Press. (2000).

[33] Young IM. De-centering deliberative democracy. In: Barker D, McAfee N, McIvor DW (eds) *Democratizing Deliberation: a Political Theory Anthology*. Dayton, OH: Kettering Foundation Press. (2006) 113–28.

[34] Scudder MF. Deliberative democracy, more than deliberation. Polit Stud. (2023) 71:238–55. doi: 10.1177/00323217211032624

[35] Habermas J. The theory of communicative action. In: Tr. T. McCarthy (eds). The Critique of Functionalist Reason. Cambridge: Polity Press (1987).

[36] Clark A Percy-Smith B. Beyond consultation: Participatory practices in everyday spaces. Child Youth Environ. (2006) 16:1–9. doi: 10.1353/cye.2006.0021

[37] Cornwall A. Making spaces, changing places: situating participation in development. Brighton: Institute of Development Studies (2002).

[38] Curtis S. Space, place and mental health. London: Routledge. (2016). doi: 10.4324/9781315610160

[39] Parr H. Mental health, public space, and the city: questions of individual and collective access. Environ Plan D: Society Space. (1997) 15:435–54. doi: 10.1068/d150435

[40] Parr H. Mental health and social space: Towards inclusionary geographies? Oxford: John Wiley and Sons. (2011).

[41] Brownlie J Spandler H. Materialities of mundane care and the art of holding one's own. In Buse, C., Martin, D., Nettleton, S. (Eds) *Materialities of Care: Encountering Health and Illness Through Artefacts and Architecture* (2018). p.14–27. doi: 10.1002/9781119499749.ch2 29464771

[42] Price A Gupta U Srivastava U Chu L. Co-production, co-education and person-centered healthcare practice. European J Person Centered Healthcare. (2019) 7:219–22.

[43] Moniz S Karia A Khalid AF Vindrola-Padros C. Stories for change: the impact of public narrative on the co-production process. Health Expect. (2023) 26:919–30. doi: 10.1111/hex.13718 36707932 PMC10010083

[44] Barnes M. Passionate participation: Emotional experiences and expressions in deliberative forums. Critic Social Policy. (2008) 28:461–81. doi: 10.1177/0261018308095280

[45] Martin GP. Public deliberation in action: Emotion, inclusion and exclusion in participatory decision making. Critic Social Policy. (2012) 32:163–83. doi: 10.1177/0261018311420276

[46] Wijnendaele BV. The politics of emotion in participatory processes of empowerment and change. Antipode. (2014) 46:266–82. doi: 10.1111/anti.12034

[47] Gardiner ME. Wild publics and grotesque symposiums: Habermas and Bakhtin on dialogue, everyday life and the public sphere. Sociol Rev. (2004) 52:28–48. doi: 10.1111/j.1467-954X.2004.00472.x

[48] Loughnane C Kelleher C Edwards C. Care full deliberation? Care work and Ireland's citizens' assembly on gender equality. Critic Social Policy. (2023) 43:697–717. doi: 10.1177/02610183231169195

[49] Meijer A Grimmelikhuijsen S Brandsma GJ. Communities of public service support: citizens engage in social learning in peer-to-peer networks. Gov Inf Q. (2012) 29:21–9. doi: 10.1016/j.giq.2011.06.004

[50] Bovaird T. Beyond engagement and participation: User and community coproduction of public services. Public Adm Rev. (2007) 67:846–60. doi: 10.1111/j.1540-6210.2007.00773.x

[51] Hovden JF. Worlds apart. On class structuration of citizens' political and public attention and engagement in an egalitarian society. Eur J Cult Pol Soc. (2023) 10:209–32. doi: 10.1080/23254823.2022.2090401

[52] McCallum SL Mikocka-Walus AA Gaughwin MD Andrews JM Turnbull DA. I'm a sick person, not a bad person': patient experiences of treatments for alcohol use disorders. Health Expect. (2016) 19:828–41. doi: 10.1111/hex.12379 26111429 PMC5152715

[53] Cairns J Nicholls J. Public Involvement in Alcohol Research. London: Alcohol Research UK. (2017).

[54] Hodge S. Competence, identity and intersubjectivity: applying Habermas's theory of communicative action to service user involvement in mental health policy making. Social Theory Health. (2005) 3:165–82. doi: 10.1057/palgrave.sth.8700055

[55] Cheng R Smith C. Engaging people with lived experience for better health outcomes: collaboration with mental health and addiction service users in research, policy, and treatment. Toronto: Ontario Ministry of Health and Long-Term Care. (2009).

[56] Neblo MA. Impassioned democracy: the roles of emotion in deliberative theory. Am Polit Sci Rev. (2020) 114:923–7. doi: 10.1017/S0003055420000210

[57] Tronto JC. Caring democracy: markets, equality, and justice. In: Caring Democracy. New York, NY: New York University Press (2013).

