# Supplementary material for: ACTIVE involvement in alcohol care: a community case study in coproduction
Source: Front Public Health. 2026 Jun 18;14:1816664. doi: 10.3389/fpubh.2026.1816664 (PMC13323507; doi:10.3389/fpubh.2026.1816664)
Supplement: Supplementary file 1 [file Data_Sheet_1.pdf]

Champs Public Health Collaborative

Working together to improve health and wellbeing in Cheshire and Merseyside

Dear Redacted

Proposed interim arrangement – Development and delivery of Patient and Public Involvement (PPI) support for the Early identification of co-occurring Alcohol and Mental Health disorders (ExAMH) Project

1. The Client appoints the Contractor to provide certain Services. The Contractor accepts that appointment.
2. The parties agree that the appointment shall be on the attached terms.
3. If you agree to the attached terms, please sign this letter, and arrange for it to be signed on behalf of the Contractor by its suitably authorised representative.

Agreed and accepted on behalf of the Contractor

Yours faithfully

REDACTED

Head of School of Nursing

[name and title of signatory]

5.4.22

Terms of this Agreement

1.	Details of the parties	
	**Client**	**Contractor**
Name	Cheshire and Merseyside Directors of Public Health	University of Central Lancashire
Address	Suite 2.2, Marwood, Riverside Park, 1 Southwood Road, Bromborough, CH62 3QX	University of Central Lancashire<br>Preston<br>PR1 2HE
Contact person:	Redacted	Redacted
Contact person's telephone number(s)	07557 191 783 / 0151 666 5144	+44 (0)1772 893884
Contact person's email:	Redacted	Redacted
Reference to the contact person of a party includes any replacement contact person as communicated to the other party from time to time		

2.	Background	
2.1	Background to this Agreement:	• The aims of the Client are (among other things) are to enable local health and care professionals to work together collaboratively for the benefit of the people of Cheshire and Merseyside.<br>• PPI support is required for the Early identification of co-occurring Alcohol and Mental Health disorders (ExAMH) Project.<br>• The Client requires the services of a suitably qualified PPI support provider to further its aims.<br>• The Contractor has certain skills and experience which can be made available to the Client to further its aims.

3.	Duration of Services	
3.1	Commencement date of the Services	24th January 2022

3.2	Expiry date of the Services	31st October 2022

**4.**	**The Services**	
4.1	The Services which the Contractor must provide the Client:	See the specification in Appendix 2.
4.2	Whether Services are to be provided for the benefit of anyone else in addition to the Client:	The delivery of PPI support for the Early identification of co-occurring Alcohol and Mental Health disorders (ExAMH) Project will benefit the Alcohol Care Team of the Royal Liverpool Hospital, and their patients. Service s Mersey Care will benefit from better integration and NHSE/I will benefit from project learning, helping them to create a scalable model.

**5.**	**Working hours etc.**	
5.1	Days on which the Contractor is expected to work	• Such hours shall be provided on such days of the week as are reasonably required by the Client and as agreed with the Contractor. .
5.2	Working hours	• Hours as agreed with the Client and the Contractor.
5.3	Approvals, notifications and other arrangements if the Contractor is to be absent (e.g. holidays, illness etc)	• The Contractor shall communicate any absence which prevents them from carrying out the required number of hours in a week (e.g., due to holidays, illness etc).

**6.**	**Location, facilities**	
6.1	Location where the Services are to be carried out:	• Services will be carried out at the Clients premises or at other venues as determined by the needs of the work being undertaken.
6.2	Provision of facilities:	• Provision of facilities and support required to provide the Services (e.g. desk, computer, access to network) will be arranged by the Contractor

7.	**Fees**	
7.1	Fees payable by the Client to the Contractor in consideration for the Services (including VAT and similar taxes):	£36k (inc. VAT) in total, split across 3 payments as follows:<br>- 28 Apr 2022 = £10,000 plus VAT<br>- 28 July 2022 = £10,000 plus VAT<br>- 28 Oct 2022 = £10,000 plus VAT
7.2	How the Fees change over time (e.g. regular increases):	No changes to Fee rates are expected during the term of the agreement.
7.3	Consequences on the Fees if the Contractor cannot provide the required services	If no services are provided as per schedule in Appendix 2, then no Fees shall be payable.

7.4 The Fees shall be paid without deduction or set-off (unless the Client has a valid and recognised judgment against the Contractor for a fixed sum, in which case that sum may be set off).

7.5 All sums due from the Client to the Contractor which are not paid on the due date shall bear interest from day to day at the annual rate of 4% over the base rate from time to time of the Council's designated bank.

8. Termination

8.1 **Termination 'for convenience':** right of a party to terminate this Agreement even if no event of default then applies to the other party:

		**Client's right**	**Contractor's right**
(a)	Whether the party may do so	It may do so.	It may do so.
(b)	Procedure to terminate	By notice to the Contact Person at any time after the date of this Agreement.	By notice to the Contact Person at any time after the date of this Agreement.
(c)	Notice period (at the end of which the termination becomes effective)	30 days after the notice is given.	30 days after the notice is given.
(d)	Any termination fee payable by the terminating party to the other party	Return of fee paid on pro-rata basis	Nil.

Appendix 1 – Rules relating to this Agreement

**9.**	**General obligations of the Contractor**	
9.1	Standards to which the Contractor must provide the Services:	The highest of the following:<br>• Standards required by Law.<br>• With reasonable skill, care and diligence.
9.2	How the Contractor must ensure they conduct themselves in providing the Services	• In a lawful, professional, honest, ethical, punctual, safe, tidy and courteous manner.<br>• Using their best available knowledge, subject to any genuine confidentiality obligations she owes to third parties.
9.3	Obligations if the Contractor has a conflict of interest in relation to any matter affecting the Client:	The Contractor must declare it in a timely and proper manner on becoming aware of it.

**10.**	**Invoicing and payment**	
10.1	When the Contractor may invoice the Client (e.g. frequency, dates, occurrence of events etc):	Invoice to be submitted by the Contractor as per the schedule below.<br>- 28 Apr 2022 = £10,000 plus VAT<br>- 28 July 2022 = £10,000 plus VAT<br>- 28 Oct 2022 = £10,000 plus VAT
10.2	Due date for payment by the Client of amounts invoiced	30 days after the invoice is issued.
10.3	Required method of payment of invoices and claims for expenses	BACS payment in the relevant nominated bank account of the Contractor.

11.	Intellectual Property	
11.1	To whom Intellectual Property arising in the course of the provision of the Services (including any goodwill attached to it) is to belong:	• To the Client or its nominee, and not to the Contractor or anyone else.<br>• Such arising Intellectual Property shall vest in the Client or its nominee instantly as it arises.<br>• The Client shall grant to the Contractor an irrevocable, non-exclusive, royalty free licence to use any Intellectual Property arising from the Contractor's provision of the Services for its academic, research and publication purposes.<br>• The Contractor will use its reasonable endeavours to ensure the Services are delivered in accordance with accepted scientific and other principles and standards, but makes no representation or warranty that any the Services will lead to any specific result or to the creation of any Intellectual Property and accepts no responsibility for any use which may be made of any Intellectual Property arising from the Services. It is therefore agreed that either party utilising such Intellectual Property is fully responsible and liable for any loss, costs, claims or demands arising from that use.<br>• Any publications arising from the Services shall be decided in accordance with normal academic practice.

12.	Exit obligations	
12.1	Obligations of the Client on termination of this Agreement:	To promptly and properly return to the Contractor any property of the Contractor then in the possession or control of Client in connection with the Services.

12.2	Obligations of the Contractor on termination of this Agreement:	To do the following in a prompt and proper manner:<br>• To return to the Client any property of the Client then in the possession or control of the Contractor. This includes, without limitation and where relevant, any files or other records, and any keys or security passes or the like.<br>• To remove from all electronic storage devices of the Contractor or the Key Personnel the following:<br>- Software belonging to or licensed by the Client.<br>- Any electronic files relevant to the Services.

**13.**	**Confidentiality**	
13.1	Type of information covered as Confidential Information of the Client:	• Information relating to the Client's business activities generally, including without limitation, its business strategies, plans, finances, operations, Personnel, products or services, research activities, customers or clients or unpublished Intellectual Property<br>• It shall be deemed to include information of third parties in relation to which the Client are under a duty of confidentiality, to the extent the Contractor knows or reasonably ought to know of that duty of confidentiality.
13.2	Exceptions to the obligations in clause 12	Where any of the following applies from time to time:<br>• Where the express or clearly implied consent of the Client is given.<br>• Where reasonably necessary in the course of providing the Services, subject to the Contractor complying with reasonable directions of the Client regarding the protection of the confidentiality of the information.<br>• Where compelled by Law (including any relevant Law relating to freedom of information) to disclose the Confidential Information, subject to the Contractor doing the following:<br>- Communicating the compelled required to the Client in a timely manner.<br>- Providing (at the Client's reasonable cost) the Client with reasonable cooperation (if requested) to enable the Client to challenge the compelled disclosure.

14.	**Miscellaneous**	
14.1	How this Agreement is to be validly amended (and no other way shall be valid, including the other conduct of the parties):	• By agreement in writing of the parties.<br>• It must be clear in the written document that it is intended to amend this Agreement.

15 **Limitation of Liability**

15.1 Subject to clauses 15.2 and 15.3 below, the entire liability of the Contractor arising under or in connection with Agreement, whether in contract, tort (including negligence), breach of statutory duty or otherwise, is limited to the value of the Fee in the aggregate.

15.2 Subject to clause 15.3 below, the Contractor shall not be liable to the Client for any indirect, special or consequential losses or damages, howsoever arising, under or in connection with this Agreement.

15.3 Nothing in this Agreement shall operate to restrict or exclude either Party's liability for death or personal injury caused by negligence or any other liability which cannot be restricted or excluded by law.

15.4 The Client undertakes to make no claim in connection with this Agreement or its subject matter against any individual employee, student, agent or appointee of the Contractor (apart from claims based on fraud or wilful misconduct).

Appendix 2 – Project Background and services to be provided

Background to the ExAMH Project

People with Alcohol Use Disorder have a higher risk of developing common mental health problems like depression and anxiety. They are more likely and more often to encounter alcohol specialist teams and GPs, rather than mainstream mental health services. In addition, they are less likely to receive treatment for their mental health disorders, for many reasons, including poor identification of these conditions. Therefore, early identification, appropriate referral, and support to take up mental health treatment would have a significant impact on mental health and alcohol addiction outcomes.

The pilot would focus firstly on services linked to Liverpool University Hospitals NHS Foundation Trust (LUFT), including the liaison mental health and IAPT services provided by Mersey Care. It is intended to improve integration between the services and facilitate improved access to mental health services at discharge from the acute hospital, or for patients already in community care.

PPI support required

It is acknowledged that the patient group under consideration for this project comprise a highly stigmatised group and can lack confidence to make meaningful contributions to large steering groups populated by authority/expert figures. Therefore, to ensure meaningful engagement for our patient group we intend to collaborate with Preston Cooperative Development Network which is active within the Preston Model for community wealth building, which includes substantial public engagement including with people from seldom heard perspectives and deprived neighbourhoods.

To do this we will provide resources to a PPI representative to ensure capacity and support to utilise these networks and the infrastructure developed within Comensus to convene a panel of individuals with lived experience of significant alcohol problems intersecting with mental health difficulties. This panel will feed into our project group and will consider the project from a PPI perspective within a protected space for free and open deliberations and contributions.

The project lead will act as a bridge between the PPI group and the project management group. The real value for PPI will be in the peer supported space where matters of comfort, ease and empowerment can be more readily supported. The panel so convened will meet in appropriate community settings, such as The Brink alcohol free bar.

The aims for PPI involvement include:

1. Co-design all patient facing documentation

2. Advise on potential impact of screening procedures from a patient and family perspective
3. Co-design and refine KPIs for the project

Objectives	Outputs	Expected completion
PPI initiation	- Initial meeting with Royal Liverpool ACT Lead<br>- Identify Project Lead<br>- Meet with ExAMH Project Team	Jan 2022
Recruitment for members of PPI group	- Utilise networks known to Comensus and the wider project team to identify 5-10 core members of PPI group. Group selection will largely be based upon relevant experience and interest, but attempt will be made to maximise diversity across group membership<br>- Convene first meeting to provide opportunities for group to feedback on project aims, processes and paperwork/assessment tools.<br>- Agree future terms of engagement for PPI group	Jan 2022 – mid Feb 2022<br><br>End Jan 2022<br><br>End Jan 2022
Agree schedule of PPI meetings	- Discuss schedule of meetings balancing project demands and capacity/availability of PPI group members. There may be an agreed division of effort in relation to specific project tasks.	End Jan 2022 – Mid Feb 2022
Development of patient facing documentation	- Workshops held to scrutinise and refine proposed documentation, and co-create any necessary new documentation	Feb 2022
Development of recommendations for screening procedures from a patient and family perspective	- Workshops held to reflect upon and refine planned screening tools	Feb – March 2022
PPI perspective and review of work in progress.	- Workshops held to reflect upon and react to issues apparent in wider project implementation. PPI group will receive reports to consider from project team<br>- Meetings with project team to facilitate two-way dialogue about project.	March 2022 – September 2022
Development of recommendations for KPI's	- PPI group coproduce salient user defined KPIs in two set piece workshops with relevant members of project team.<br><br>- PPI report produced.	September 2022<br><br>Sept- Oct 2022
Production of short film(s) to celebrate key aspects of the project.	- Contracted Film maker, David McCollom, an ex-service user with relevant lived experience works with PPI group and select members of project team to produce at least one short film. Other filmic outputs may be considered depending on nature of inputs/outputs of project and various contributions to the creative process	April 2022 – October 2022

Champs
Public Health
Collaborative

Working together to improve health and wellbeing in Cheshire and Merseyside
