# Supplementary material for: ACTIVE involvement in alcohol care: a community case study in coproduction
Source: Front Public Health. 2026 Jun 18;14:1816664. doi: 10.3389/fpubh.2026.1816664 (PMC13323507; doi:10.3389/fpubh.2026.1816664)
Supplement: Supplementary file 2 [file Data_Sheet_2.pdf]

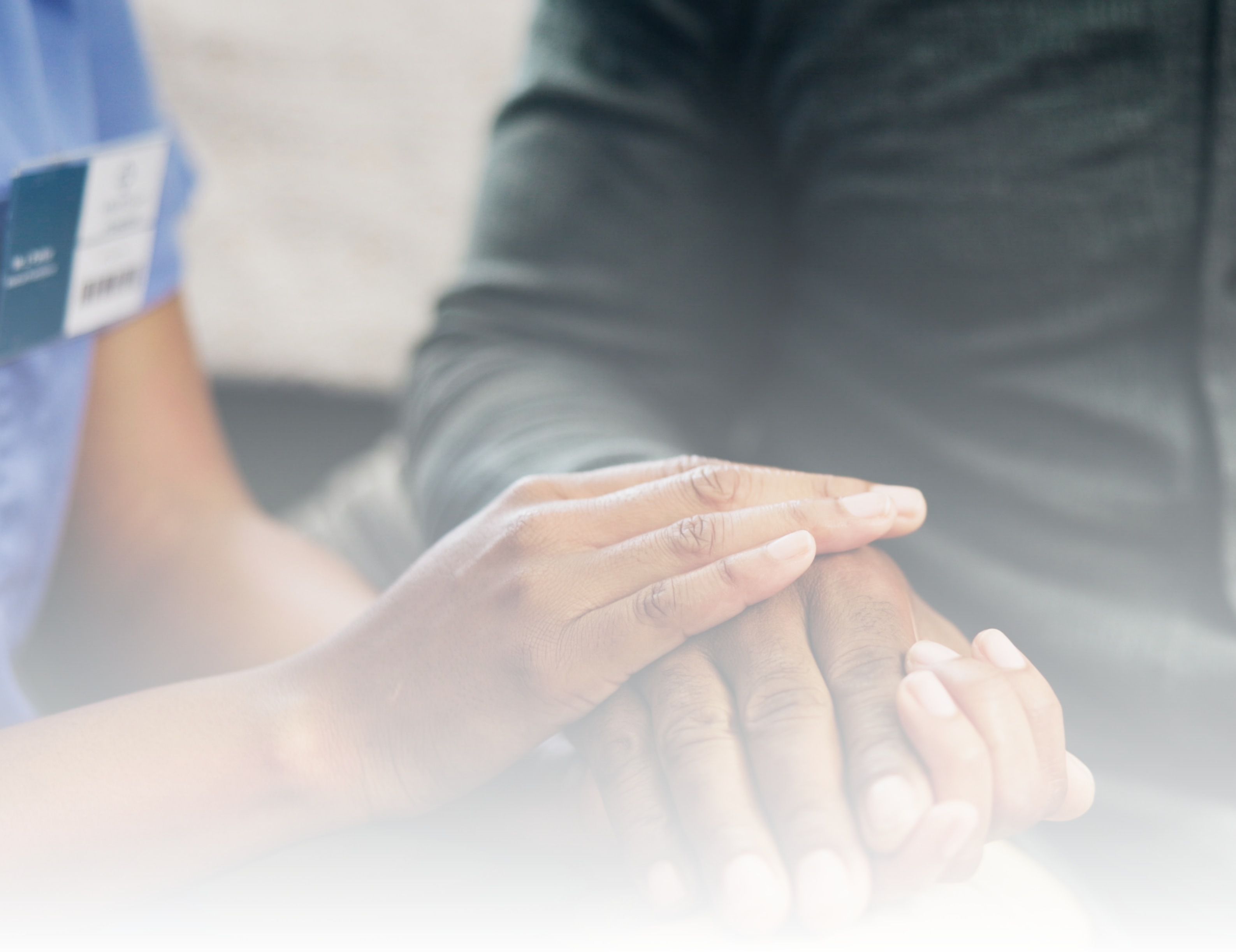

Programme for Alcohol Care and Treatment (PROACT)

A Practice Learning Guide:
Clinical Placement with an Alcohol Care Team in Cheshire and Merseyside

Champs Public Health Collaborative

PROACT
PROGRAMME FOR ALCOHOL CARE AND TREATMENT

CONTENTS

Section	Title	Page
1	Introduction: Welcome to our team	2
	The purpose of the practice guide	2
	What does an ACT do?	3
	Aims for your time with the ACT	3
	Learning objectives for your time with the team	4
	Raising concerns	4
2	Getting the most from your experience	5
3	Learning	7
	Some useful facts and figures	7
	Screening	8
	Interventions to achieve abstinence or alcohol reduction	15
	Families	17
	Patient and Public Involvement	18
4	Your development	19
	Skills and competence	19
	Education and training	19
5	Going home checklist	20
6	Appendix	21
7	References	30

WELCOME TO OUR TEAM

PURPOSE OF THIS PRACTICE GUIDE

This practice learning guide was developed by our Senior Nurse Advisory Panel (SNAP) in partnership with our ACTIVE PPI group which has representation from across Cheshire and Merseyside. It will provide you with important guidance enabling you to maximise and enjoy your learning opportunities in the clinical practice of caring for people who present to an acute hospital with an Alcohol Use Disorder (AUD).

It is important to remember that this is an additional tool to be used in conjunction with your organisation's guidelines, governance, standards and procedures. Practice learning is an opportunity for you to develop the resilience, skills, knowledge and caring needed to provide evidence-based care as part of an interprofessional team[1].

To date there is no standardised approach to supporting clinical placements within Alcohol Care Teams (ACTs). This guide is designed to provide a structure to achieve consistency and prevent variation across our network. Caring is a lifelong career with potential for progression. We offer you the opportunity to nurse/care for this highly complex group of patients who have come to harm due to drinking alcohol. You will meet patients along a spectrum of AUD's and co-existing morbidities.

As a student at the start of your career, you may struggle with a chaotic system of competing interests, theories and approaches to care, all of which distract from your ability to learn how to provide safe, compassionate and streamlined patient care. Our unique approach is derived from us building a network of nurses, carers, other professionals, commissioners and, most importantly, our patient and public involvement group (ACTIVE), who asked us to be different. They asked us to demystify, explain and provide a workable structure for us to build a community of skilled, competent, compassionate, engaged and supportive alcohol care specialists. By including our students in this process we hope to future proof any improvements we make, by supporting and building a future workforce, and that may well include you! This will ensure that any improvements we make are sustainable.

There follows a list of abbreviations and acronyms in common use in our service and this handbook. These terms and concepts are useful in organising services and obtaining resources, but our ACTIVE group have urged us to be careful and sensitive in using these in everyday practice. This means being aware of some dangers of attaching labels to people - treating patients as people first and foremost, regardless of what labels they carry.

ABBREVIATIONS

**AAW**	Acute Alcohol Withdrawal	**ED**	Emergency Department
**ACT**	Alcohol Care Team	**FE**	Fibroelastography
**ACTIVE**	Alcohol Care Team InVolvement and Engagement Group	**FRAMES**	A protocol for delivery of BI
**AD**	Alcohol Dependence	**GAD 7**	Generalized Anxiety Disorder Questionnaire
**ARCI**	Alcohol Related Cognitive Impairment	**LD**	Liver Disease
**ASN**	Alcohol Specialist Nurse	**MAW**	Medically Assisted Withdrawal "detox"
**AUD**	Alcohol Use Disorder	**MH**	Mental Health
**AUDIT**	Alcohol Use Disorders Identification Tool	**MoCA©**	Montreal Cognitive Assessment Tool
**BA**	Brief Advice	**NICE**	The National Institute for Health and Care Excellence
**BI**	Brief Interventions	**PHQ 2 and 9**	Patient Health Questionnaire
**CG**	Clinical Guideline	**PPI**	Patient and Public Involvement
**CIWA**	Clinical Institute Withdrawal Assessment for Alcohol	**PROACT**	Programme for Alcohol Care and Treatment

WHAT DOES AN ACT DO?

Alcohol Care Teams exist to provide compassionate care and support for individuals with alcohol problems and their families. The NHS Long Term Plan states that alcohol care teams (ACTs) primarily provide specialist expertise and interventions for alcohol dependent patients and those presenting with acute intoxication or other alcohol-related complications, attending A&E or admitted as inpatients across most departments of the acute hospital.

ACTs have been shaped by a number of historical developments. The first job description explaining the role of an Alcohol Specialist Nurse (ASN) was published by the Royal College of Physicians (RCP) in 1990[2]. Building on this role, acute UK hospitals began to develop teams of nurses aimed at improving the outcomes of patients with an AUD. This led to the development of a formalised approach were Alcohol Care Teams (ACTs) were recognised as a speciality.

In 2010 ACTs were defined in a joint publication by the British Society of Gastroenterology, Alcohol Health Alliance UK and the British Association for the Study of the Liver[3], which made key evidence-based recommendations, outlining core key functions of an ACT.

In the subsequent decades this has evolved.

An ACT will typically provide some or all of the interventions below:

1. Case identification/alcohol identification and brief advice (IBA).
2. Comprehensive alcohol assessment.
3. Specialist nursing and medical care planning.
4. Management of medically-assisted alcohol withdrawal (MAW).
5. Provision of psychosocial interventions.
6. Planning safe discharge, including referral to community services.
7. Clinical leadership by a senior clinician with dedicated time for the team.
8. Provision of trust-wide education and training in relation to alcohol.

In the subsequent decades this has evolved.
In 2019 the government published its Long Term Plan https://www.longtermplan.nhs.uk/publication/nhs-long-term-plan/, and as part of this, the NHS Prevention Programme supported the establishment ACTs in hospitals deemed to be at greatest need.

AIMS FOR YOUR TIME WITH THE ACT

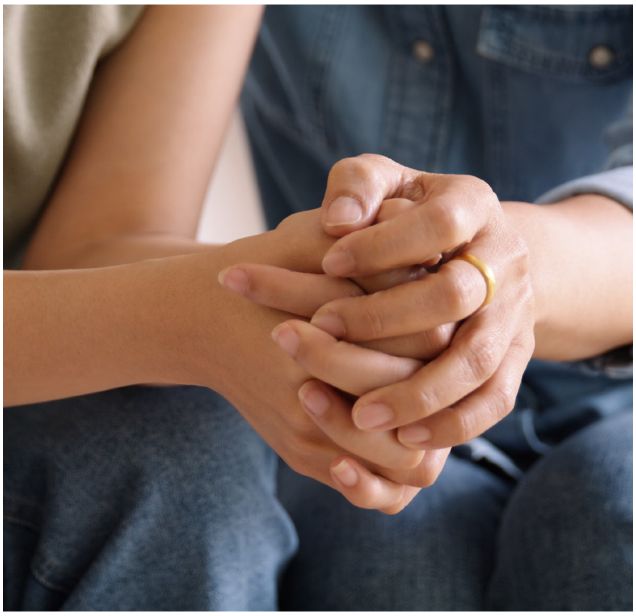

- Provide a safe environment for you to learn about our patient group.
- Provide opportunities for you to learn about and understand the interdependencies of different care providers involved in our patient pathways. In Cheshire and Merseyside, all of these services are joined together in a mutually supportive network called PROACT
- Enable you to use the experts in the ACT to guide you in critically evaluating the evidence-based practice and procedures employed by this service.
- Make this an enjoyable and enhancing experience that will contribute to your personal development.

LEARNING OBJECTIVES FOR YOUR TIME WITH THE TEAM

- Understand the scale of alcohol harms.
- Know how to assess for alcohol-related risk: screening tool use.
- Know how to categorise alcohol-related risk: screening tool interpretation.
- Understand interventions available for medium and high risk drinkers
  - Understand the basic mechanisms of Acute Alcohol Withdrawal.
  - Improve your confidence in discussing alcohol with patients, i.e. taking an alcohol history in a therapeutic way.
  - Improve knowledge and skills in the interpretation of diagnostic results and how these will guide interventions and treatments.
  - Improve your skills in identifying Acute Alcohol Withdrawal.
  - Improve knowledge of pharmacological treatments for Acute Alcohol Withdrawal.
- Improve your understanding of how alcohol can affect individuals and their significant others, including:
  - Physically
  - Mentally
  - Emotionally and spiritually
  - Socially
  - Economically
  - Develop confidence and understanding in how to relate to individuals and their families in ways that reflect a better understanding of the impact of alcohol in their lives.

RAISING CONCERNS

- You may see something in practice that concerns you. For nurses, the NMC (2019b) provides guidance for raising concerns, but all services will have a policy to assist you so make sure you know where to access this. Should you have any concerns during your working day, discuss your worries with the team first.
- Do not wait to raise any urgent concerns.

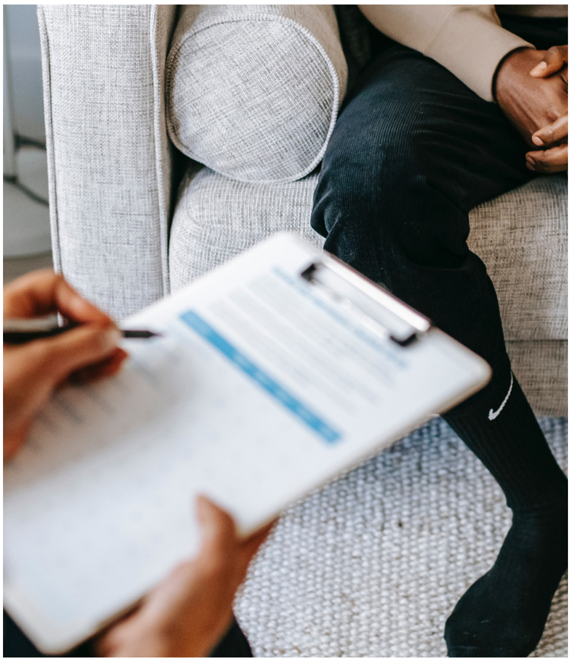

2. GETTING THE MOST FROM YOUR EXPERIENCE

It might be useful to use the simple model below to guide your experience. Ask yourself:

Knowing and understanding

What do I know now?

What do I need to know?

How best can I learn?

Thinking and Feeling

How do I ensure I feel safe?

Do I feel more confident?

Am I enjoying this?

Doing

Am I learning new skills?

Can I apply them to my practice?

Can I reflect on the value and impact of practice for patients?

Here are some questions you could ask the team at the start of the placement

- What do you expect of me during my time with the team?
- Are there procedures specific to this speciality that I might not have come across before?
- What are the formal and informal lines of communication?

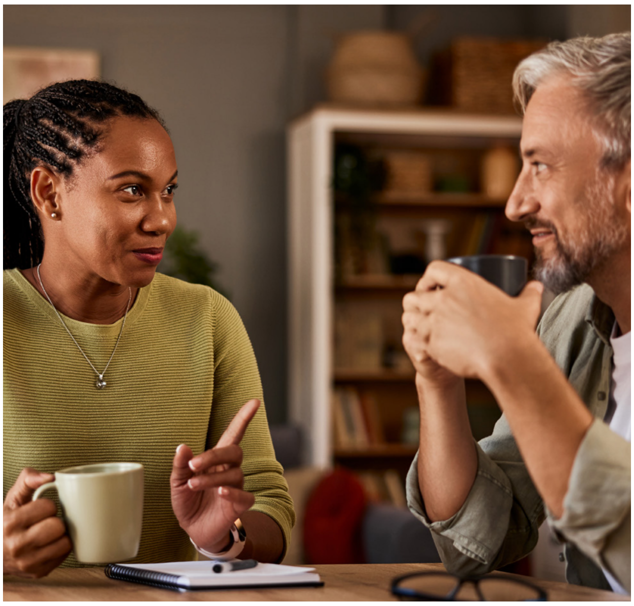

Plan your journey with your senior nurse. Use this as your own personal plan:

**START** Plan	
**MIDDLE** What do I need to do	
**END** Evaluate and refelct	

3. LEARNING

SOME USEFUL FACTS AND FIGURES

WHAT IS ALCOHOL?

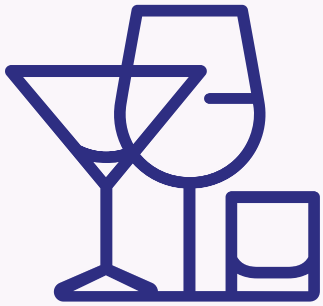

- It is a by-product of fermentation of grains and fruits
- Acts directly on the central nervous system
- It has been around for twelve thousand years
- It is a depressant
- It is mind altering
- It has been used as medicine, as a staple fluid, currency and religious ceremonies

FUN FACTS

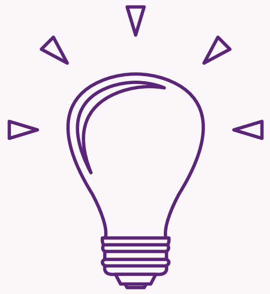

DID YOU KNOW?

- An ancient Chinese law from 1116 BC states that alcohol use was required by heaven
- Early Egyptian writings urge mothers to send their children to school with plenty of bread and beer
- In the Middle Ages monasteries produced the majority of alcohol, it was known as Aqua Vitae which meant ‘water of life’
- The word ‘toast’ used to mark special occasions derives from an ancient Roman custom of dipping bread into wine at formal gatherings

Alcohol is a simple molecule. There are over a hundred different types of alcohol including:

- Ethanol = C2 H5 OH
- Methanol = CH3 OH
- Isopropyl = (Ch3)2 CH2 OH

Alcohol is classified as a central nervous system (CNS) depressant. It:

- Contains no vitamins, minerals etc.
- Contains 210 calories/oz
- Requires no digestion
- Once in the system it stays until metabolized
- This makes it quite a unique molecule

DETECTING ALCOHOL USE DISORDER

From a purely clinical perspective, the detection of AUD is crucial to improving people's lives and clinical outcomes. Consistent, reliable screening is essential, but this must be achieved in a context of high quality inter-personal relationships. Building this relationship of respect and trust is integral so individuals feel safe to have an honest conversation without feeling judged. A key message from the ACTIVE PPI group stresses the importance of these relational skills. The initial first impression is really important.

> ‘‘ *As a care giver you should be leading with a compassionate, deeply empathic communication style that treats people with dignity. This will build rapport and trust and make all of our interventions more successful.’*
>
> (ACTIVE member)

Screening can be stratified into three main forms of assessment:

1. Detecting AUD in the absence of physical dependence.

2. Detecting alcohol dependence and stratifying severity and level of risk.

3. Detecting co-existing or co-morbid conditions (for example, liver disease, mental health)

The National Institute for Health and Care Excellence (NICE) PH24[4] recommendation is

> *“ Complete a validated alcohol questionnaire with the adults being screened. Alternatively, if they are competent enough, ask them to fill it in themselves. Use AUDIT to decide whether to offer them a brief intervention (and, if so, what type) or whether to make a referral. …Screening tools should be appropriate to the setting…”*

The Alcohol Use Disorders Identification Tool (AUDIT) questionnaire consists of ten questions aimed at eliciting alcohol consumption, drinking behaviour and alcohol-related problems. The aim is to detect ‘hazardous’ and ‘at risk’ drinkers, and is scored on a scale of 1 to 40. A score of 8 or more indicates the requirement for further intervention, and the likelihood of experiencing social problems from drinking[5&6].

The authors of the questionnaire have shown that it takes just two minutes to administer, with a sensitivity of 92% and specificity of 94% in detecting hazardous drinkers[5] which is higher than any other tool or biochemical marker[7]. There is also a shortened version of this tool called AUDIT-C which consists of three 3 of the original questions. AUDIT-C takes less time to administer and has been shown to be reliable with a sensitivity of 78%[8].

ALCOHOL USE DISORDERS IDENTIFICATION TOOL (AUDIT) IN CLINICAL PRACTICE

A guide to use and interpretation for Alcohol Support Nurses.

- ✓ Score = <5 no risk
- ✓ >5 <8 minimal risk
- ✓ >8 < 15 risky drinking amenable to a brief intervention (BI)
- ✓ 15 high risk and potential dependence EBI = SADQ

STEP 1 (AUDIT-C)

a) AUDIT-C consists of the first 3 questions of the full AUDIT tool.

b) A score of 5 or less indicates no identified risk. However, use the opportunity to provide brief advice, possibly provide a leaflet on units, and thank the patient for their time.

c) If the score is greater than 5, complete the rest of he AUDIT questions.

QUESTIONS	SCORING SYSTEM					
	**0**	**1**	**2**	**3**	**4**	**Score**
How often do you have a drink containing alcohol?	N	1 month	2-4 month	2-3 week	>4 week	
How many units of alcohol do you drink on a typical day when you are drinking?	1-2	3-4	5-6	7-9	10+	
How often have you had 6 or more units if female, or 8 or more if male, on a single occasion in the last year?	N	Less than monthly	monthly	weekly	daily	
					**Total**	

STEP 2 (AUDIT)

a) Complete the full **AUDIT**; if the score is less than **8**, use the opportunity to provide brief advice, possibly provide a leaflet on units and thank the patient for their time.

b) If the score is greater than **8** but less than **15**, deliver a brief intervention, and offer the patient follow-up in the community for extra support if needed.

c) If the patient score is greater than **15**, assess for dependence utilising the Severity of Alcohol Dependence Questionnaire score (SADQ)

d) Any positive score on SADQ please refer to an ASN for assessment. If the score is greater than **30** the ASN should be bleeped for an urgent assessment to be performed.

Deliver Brief Advice

QUESTIONS	SCORING SYSTEM					
	0	1	2	3	4	Score
How often during the last year have you found that you were not able to stop drinking once you had started?	Never	Less than monthly	monthly	weekly	Daily or almost daily	
How often during the last year have you failed to do what was normally expected from you because of your drinking?	Never	Less than monthly	monthly	weekly	Daily or almost daily	
How often during the last year have you needed an alcoholic drink in the morning to get yourself going after a heavy drinking session?	Never	Less than monthly	monthly	weekly	Daily or almost daily	
How often during the last year have you had a feeling of guilt or remorse after drinking?	Never	Less than monthly	monthly	weekly	Daily or almost daily	

QUESTIONS	SCORING SYSTEM					
	0	1	2	3	4	Score
How often during the last your have you been unable to remember what happened the night before because you had been drinking?	Never	Less than monthly	monthly	Weekly	Daily or almost daily	
Have you or somebody else been injured as a result of your drinking?	No		Yes, but not in the last year		Yes, during the last year	
Has a relative or friend, doctor or other health worker been concerned about your drinking or suggested that you cut down?	No		Yes, but not in the last year		Yes, during the last year	
					**Total**	

STRATIFYING RISK AND DETECTING DEPENDENCE

Several approaches have been developed to assist in quantifying the levels of alcohol dependence. For example, the Severity of Alcohol Dependence Questionnaire (SADQ), measures severity of dependence on a 0 to 60 point scale, a score of 30 or above indicating severe dependence[9] and can be administered prior to any manifestation of symptoms. It is therefore a useful tool in the implementation of prophylactic treatment (Appendix 1).

Additionally, tools to map the course and severity of alcohol withdrawal symptoms (AWS) in the clinical setting have been developed. In particular the Clinical Institute Withdrawal Assessment for Alcohol (CIWA versions Ar and AD) Scale[10 & 11] (Appendix 2) and the Glasgow Modified Alcohol Withdrawal Scale (GMAWS) (Appendix 3)[12].

It is important to note that many services have adapted the GMAWS dosing regimen to optimise compliance with usual and historic prescribing practice in their organisation.

The National Institute for Health and Care Excellence (NICE) CG100[13] recommendation is

> *“ Follow locally specified protocols to assess and monitor patients in acute alcohol withdrawal. Consider using a tool (such as the Clinical Institute Withdrawal Assessment - Alcohol, revised [CIWA-Ar] scale)[11] as an adjunct to clinical Judgement.”*

TREATMENT

The overall and most important objective in treating the alcohol dependent patient is to ensure safe, timely, appropriate and effective care. This can only be achieved through accurate and timely assessment and detection of co-morbid conditions that may effect the treatment choices, presence and severity of AAW.

AIMS OF MEDICAL MANAGEMENT OF ACUTE ALCOHOL WITHDRAWAL (AAW)

It is important to avoid a) inadequate treatment, which may lead to Delirium Tremens or seizures and b) over treatment, which may lead to over sedation and respiratory depression.

Early detection and prompt initiation of treatment is crucial as untreated AAW can progress to delirium tremens, which has been shown to be fatal in 15-20% of untreated patients.

If untreated, death may result from respiratory and cardiovascular collapse or cardiac arrhythmias. Patients most at risk are those with either a high fever (>104°F/39.9°C), tachycardia, or dehydration, and an associated illness (e.g. pneumonia, decompensated liver disease, or pancreatitis), general debility or where the diagnosis is delayed.

However, appropriate management reduces mortality to around 1%. In most cases this can be achieved with oral benzodiazepines.

Because of the psychological impact of detoxification, planning and coordination with alcohol follow-up services is essential. Good nursing care in a well-lit, cool environment has been shown to reduce the impact of sensory deprivation on the confused patient and, as such, is a crucial part of the treatment plan.

Alcohol consumption results in the brain adjusting function in the presence of alcohol. If the intake of alcohol is stopped, the blood-alcohol level decreases but the brain remains in a hyper-excited state, which leads to the withdrawal syndrome.

Table 1: Features of alcohol withdrawal syndrome

COMMON FEATURES		LESS-COMMON
Hand tremor	Sweating, Flushing	Arrhythmias
Minor hallucinations	Tachycardia	Hypertension
Insomnia	Convulsions	Paraesthesiae
Anxiety, Agitation,	Nausea, Vomiting,	Hepatic dysfunction
Confusion, Disorientation	Anorexia, Diarrhoea	Suicidal ideation

Benzodiazepines are central to the management of alcohol withdrawal and have the following important properties: sedative, anxiolytic, anticonvulsant, cross-tolerant with alcohol, and do not induce liver enzymes.

The National Institute for Health and Care Excellence (NICE)[13] caution that

> *" People with decompensated liver disease who are being treated for acute alcohol withdrawal should be offered advice from a healthcare professional experienced in the management of patients with liver disease." [CG100].*

This includes the careful titration of benzodiazepines to mitigate the effects of organ disfunction.

EQUIVALENT DOSES OF BENZODIAZEPINES

It is difficult to determine an exact equivalent dose of benzodiazepines. Differences in pharmacokinetic parameters between benzodiazepines are significant. Also, research has not consistently identified equivalent dosages between benzodiazepines. Using a range of +/- 25% the table below is a guide and should not replace clinical decision making

Table 2: Conversion dose equivalence of benzodiazepines.

DRUG	EQUIVALENT DOSE	*RANGE +/- 25%
Chlordiazepoxide	10mgs	
Lorazepam	0.5 mg	0.3 - 0.5 mg
Diazepam	2 mg	1.5 - 2.5 mg
Oxazepam	5 mg	4.5 - 7.5 mg

*The actual equivalent dosage could be at the lower or upper end of this range.

DETECTING CO-EXISTING ORCO-MORBID CONDITIONS

We have provided three examples of commonly occurring, co-morbid conditions (liver disease, mental health and cognitive impairment), however it is important to remember there are many more

Liver Disease

Liver disease is silent for most in the pre-cirrhosis phase (fibrosis) and in early cirrhosis. There may be no signs or symptoms associated with early cirrhosis, only the conditions that result in the fibrosis.

Cirrhosis is known to affect sociodemographic groups differently. The highest rates of liver disease are found in the most deprived areas of the UK.

Liver Function Tests (LFTs) and alcohol

All patients identified as having an Alcohol Use Disorder and assessed by the ACT will have a liver screen including:

- **GGT** – recommended by NICE as a form of biofeedback to show effects of reduction of alcohol intake. Sensitivity and specificity are low – depending on the population between 50 to 60%.
- There are three tests that measure inflammation, or damage to liver cells and are not testing function. These are:
  - **ALT** - released in the blood stream when hepatocytes are damaged
  - **AST** - released in the blood stream when hepatocytes are damaged **AST:ALT** ratio is useful for alcohol aetiology (2:1)
  - **Alk Phosphate** – not specific for the liver, mainly in the lining of the biliary ducts, but like ALT and AST a sign of liver damage
- There are three tests that are needed to establish actual liver damage and prognosis using the Child Pugh scoring system:
  - **Bilirubin** – if this is raised seek advice from hepatologist, if >50, refer to a hepatologist.
  - **Albumin** – main protein synthesised by the liver.
  - **INR or PT** - raised. Many causes so may be indicative of impaired liver synthetic function.

Fibro Elastography (FE)

The FE score is useful as an objective measure for comparisons over time[14], and perhaps more importantly is a useful clinical tool for patient feedback in motivation to reduce/stop drinking. It provides objective evidence for the positive effects of alcohol reduced drinking[16]. As active drinking can cause results to be erroneous it is important to explain this to the patient and arrange repeat scans as follows;

- Normal <7 no repeat
- Abnormal >7<15 possiblefibrosis degree – stop drinking repeat in 12 months
- >15 stop drinking – repeat 3 months

SCORING CARD

CORRELATION BETWEEN LIVER STIFFNESS (kPa) & FIBROSIS STAGE

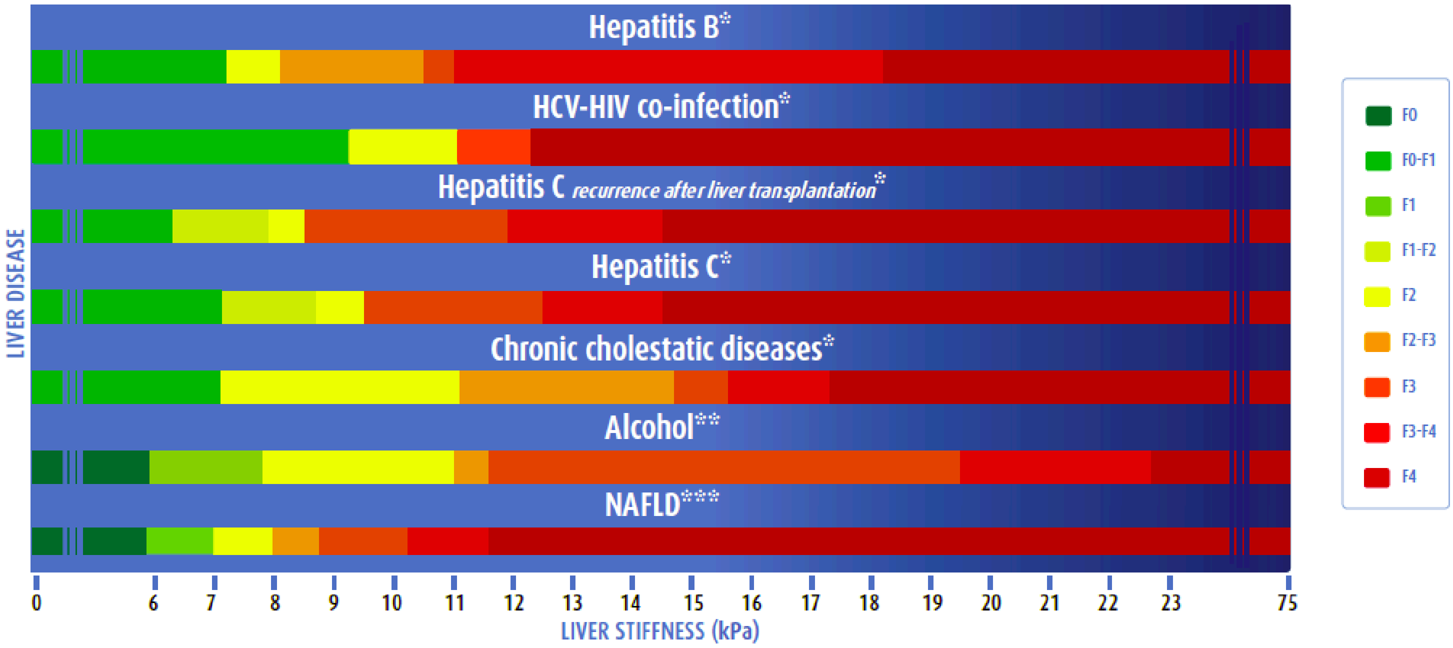

ALCOHOL RELATED COGNITIVE IMPAIRMENT (ARCI)

ARCI often presents not too dissimilar to dementia. Individuals may present with problems in: memory and organising, planning and executing tasks; insight and safety awareness and the loss of social inhibitions. If the use of alcohol continues, their cognition and functional ability continuesto deteriorate.

They are unable to manage their own day-to-day life and often present as self-neglecting and in crisis from a medical and social perspective. Therefore, detection is at the earliest opportunity is crucial to optimising potentialfor recovery[16].

A useful screening tool is the Montreal Cognitive Assessment Tool (MoCA©)[17] (Appendix 4).

“ The families of people with ARCI face numerous challenges in caring for their loved ones. It is important that families are supported in this caring role”. (ACTIVE member)

CO-OCCURRING MENTAL HEALTH

The Department of Health issued the following definition of co-occurring mental health and drug/alcohol use conditions':

> *" The term 'co-occurring mental health and drug/alcohol use conditions' covers a broad spectrum of mental health and substance use problems that an individual may experience concurrently. The nature of the relationship between these two conditions is complex. Possible mechanisms include: Substance use worsening or altering the course of a psychiatric illness; Intoxication and/or substance dependence leading to psychological symptoms; Substance use and/or withdrawal leading to psychiatric symptoms or illnesses."*

The nature of the relationship between these two conditions is complex and is typically a two-way street. Possible mechanisms by which problems with alcohol can interact with mental health problems include: Existing mental health issues can often lead people to use alcohol in excess, for instance self-medicating distress;

> *" I sometimes drank to forget. Other times I was trying to cope with anxiety or change the way I felt. To check out of my reality" (ACTIVE member)*

Aims of screening should be:

1. Early identification of depression/anxiety/psychosis in people with alcohol use disorders.
2. Greater recognition and referral for treatment of mental health conditions. ". Previously people have found that their co-existing mental health and alcohol needs haven't been met by services, or that they bounced between different services" (ACTIVE).
3. Improved clinical outcomes for alcohol use disorders and mental health conditions.

Useful tools that can be used to detect mental health issues are the Patient Health Questionnaire (PHQ 2 and PHQ7) and Generalized Anxiety Disorder Questionnaire (GAD-7) which are explained in (Appendix 4).

INTERVENTIONS TO ACHIEVE ABSTINENCE OR ALCOHOL REDUCTION

There is a very large body of research evidence supporting Brief Interventions (BI) in primary care including 56 controlled trials[19].

For every eight people who receive simple alcohol advice, one will reduce their drinking to within lower-risk levels. This compares favourably with smoking where only one in twenty will act on the advice given20. Patients who received BI in emergency departments (ED) made 50% fewer visits to the ED during the following 12 months[21&22]

A widely utilised approach to having a safe and honest conversation about the consequences of drinking alcohol is the FRAMES[23] model.

FRAMES

	DESCRIPTOR	WHY NOT ADD YOUR OWN NOTES ON EXAMPLES YOU HAVE OBSERVED
Feedback	Provides feedback on the patient's risk of alcohol problems.	
Responsibility	The individual is responsible for change.	
Advice	Advises reduction or gives explicit direction to change.	
Menu	Provides a variety of options for change.	
Empathy	Emphasises a warm, reflective and understanding approach.	
Self-efficacy	Encourages optimism about changing behaviour.	

The FRAMES model is a method used to deliver brief interventions which have been shown to be highly effective approach to helping people with AUD reduce their drinking[24], and can also be helpful in supporting dependent patients to engage in treatments toward abstinence[25].

Psychological approaches aimed at preventing relapse also include:

- Motivational enhancement therapy (MET).
- Cognitive behavioural therapy (CBT).
- Group and family-based therapies.
- Community-based and residential rehabilitation programmes.
- Interventions promoting social support and integration.
- Social behaviour and network therapy (SBNT).
- 12-step facilitation (TSF).

“ All of these interventions are improved by basic human relationship skills. Use of compassionate and empathic communication skills lead to better treatment outcomes. Try to put yourself in the shoes of the person in front of you and ask yourself: how would I feel How would I like to be treated? People, regardless of their health needs, wish to be treated with kindness and love, and will notice if this seems lacking”. (ACTIVE member)

Pharmacotherapy: Important things to consider and discuss with patients

Medicines are an important and useful adjunct to psychological approaches, however it is important to consider:

- Many patients expect their life to be perfect once they stop – sadly it isn't. They still have problems at home, they still hate their job etc.
- Psychosocial treatments (should) help them deal with this reality better and reduce the risk of relapsing.
- So – pharmacotherapies are important, but they must be given correctly, and the patient needs to be monitored regularly. In addition, pharmacotherapies can be used to enhance the efficacy of psychosocial treatments and vice versa.

Table 3. Pharmacotherapy: most commonly used medicines

MEDICATION	INDICATION	AIM
Acamprosate	Relieves craving	Abstinence
Nalmefene	Relieves craving	Alcohol reduction
Naltrexone	Relieves craving	Abstinence
Baclofen	Relieves craving	Abstinence
Disulfiram	Aversive therapy	Total alcohol avoidance

FAMILIES

Family socialisation processes have been shown to have a significant influence on a variety of health-related behaviours. Indeed, family functioning and AUD are inextricably connected. It is important to consider that families are very often harmed psychologically by their loved ones, but also that families that support their loved ones show substantial improvements in both the recovery of their loved ones and family cohesion and functioning. With help and support from services, families may be our greatest asset to helping our patient achieve their goals. Family members or significant others can successfully motivate a person with AUD to initiate changes in drinking or to seek AUD treatment. During recovery, family members can provide active support.

However, it is also important to respect the patient's wishes and rights to confidentiality. They may not wish to involve family in their care, indeed some families may be a risk to the patient. The message here is ***"listen to the patient and act always in accordance with their wishes"***.

The statistics on alcohol-related harms are stark:

- Around 18% of children 'in need' are affected by someone else's AUD.
- Parental alcohol misuse features in 37% of cases on the child protection register.
- 48% of convicted domestic abuse perpetrators had history of alcohol dependence and 73% had consumed alcohol prior to the event.

" As a relative, I have found that the regular connection I have with the ACT through being involved in clinic appointments is invaluable and so very much appreciated. I see this as making a sustained impact on my sister's progress" (ACTIVE member).

PATIENT AND PUBLIC INVOLVEMENT (PPI)

This is one of a number of initiatives aimed at placing patients at the centre of their care. Patient and public involvement is not only good practice, it is also enshrined in the Health and Social Care Act 2001 (section 11).

This is one of a number of initiatives aimed at placing patients at the centre of their care. Patient and public involvement is not only good practice, it is also enshrined in the Health and Social Care Act 2001 (section 11).

The ACTIVE PPI (patient and public involvement) group was formed in 2022, in response to a need for lived experience involvement in the shaping of a new service for people who have problems with alcohol and mental health. It has long been recognised that these people are typically failed by both alcohol and mental health services, often bouncing between the two or falling into the gap between. The Early Identification of Co-occurring Mental health and Alcohol Issues (ExAMH) pilot project was commissioned by Cheshire and Merseyside Public Health team (CHAMPS) and the ACTIVE PPI group invited to help design and consult on the new service. The success of this collaboration led to ACTIVE being further commissioned to provide ongoing PPI across the whole PROACT network of services across the region. Members of the group are committed to improvement and helping others as one person explains:

“ My involvement in ACTIVE is driven by my desire for future generations of patients to be in services that have learnt something from my experiences so that the way they support people is improved. I want people not to have to be dishonest about where they are at with their alcohol use for fear of being excluded from services or, like in my case, worrying about whether my child might be taken off me. For me, this would have made me considerably worse and I would have continued drinking or even thought about ending it all. Now I feel like I have an opportunity to make a difference and speak directly to people in the care teams. We now have a real voice to make changes.”
(ACTIVE member)

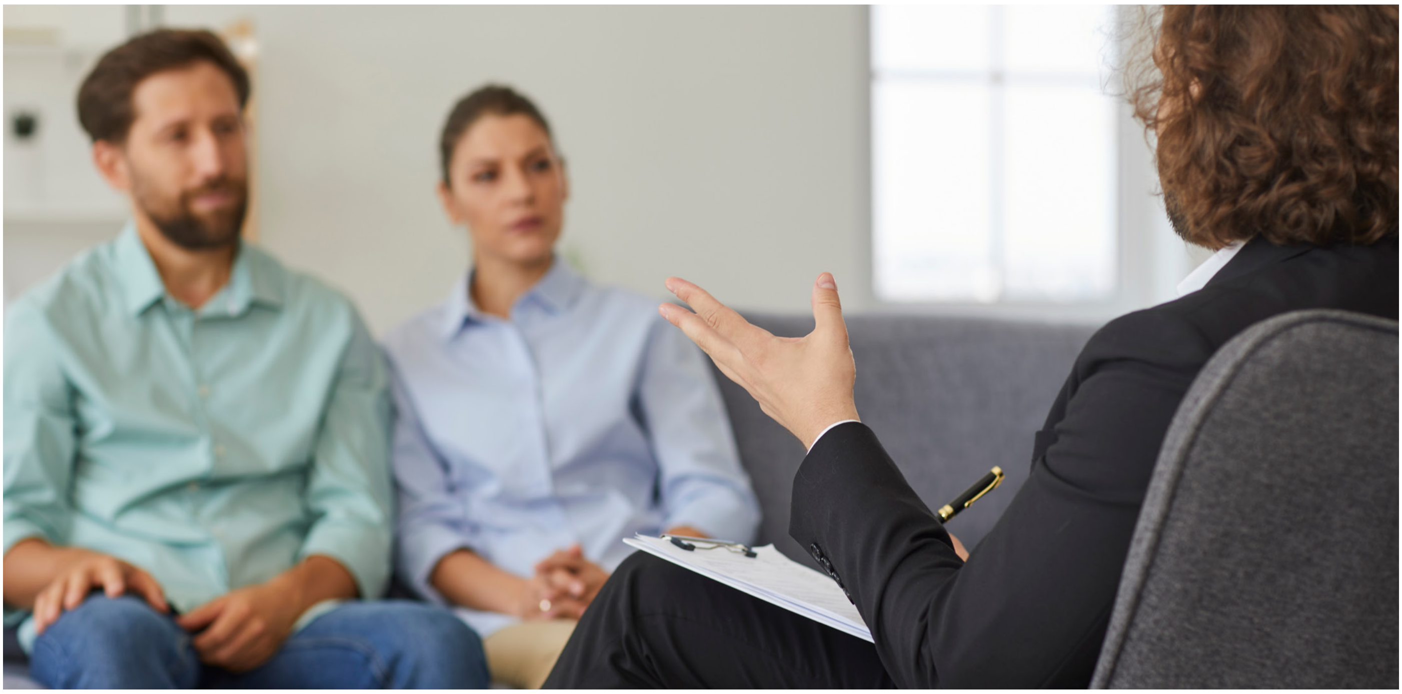

4. YOUR DEVELOPMENT

SKILLS AND COMPETENCE

We have developed the PROACT Competencies Framework which describes the attitudes, skills, and knowledge required to deliver distinct interventions shown to optimise the care of our patients.

In the Competency Framework you will find guidance for learning around 9 clinical competencies. Use this alongside this guide.

- Competency 1: The nurse/patient relationship - communicating with families and Multidisciplinary Teams (MDT).
- Competency 2: Equality and diversity.
- Competency 3: Alcohol screening and detection of alcohol use disorders (NICE PH 24).
- Competency 4: Assessment and management of the alcohol dependent patient and Alcohol Withdrawal Syndrome (NICE CG 100 and CG115).
- Competency 5: Brief interventions (NICE PH24).
- Competency 6: Non-medical prescribing.
- Competency 7: Detection and management of Wernicke's Encephalopathy and Alcohol Related Brain Injury (cognitive impairment).
- Competency 8: Detection and management of Alcohol Related Liver Disease.
- Competency 9: Detection and management of dual diagnosis patients.

EDUCATION AND TRAINING

During your time with us you will have the opportunity to attend our Regional Teaching Programme which will provide you with theory to underpin the practical element you will experience in practice.

The Alcohol Care Team has an ethos of lifelong learning which we hope you embrace (NMC 2018). There is bespoke training available online, you should speak to your mentor about organising this whilst you are on placement. Should you consider a career specialising in alcohol care, please speak to your mentors for more information on how we can support you with this.

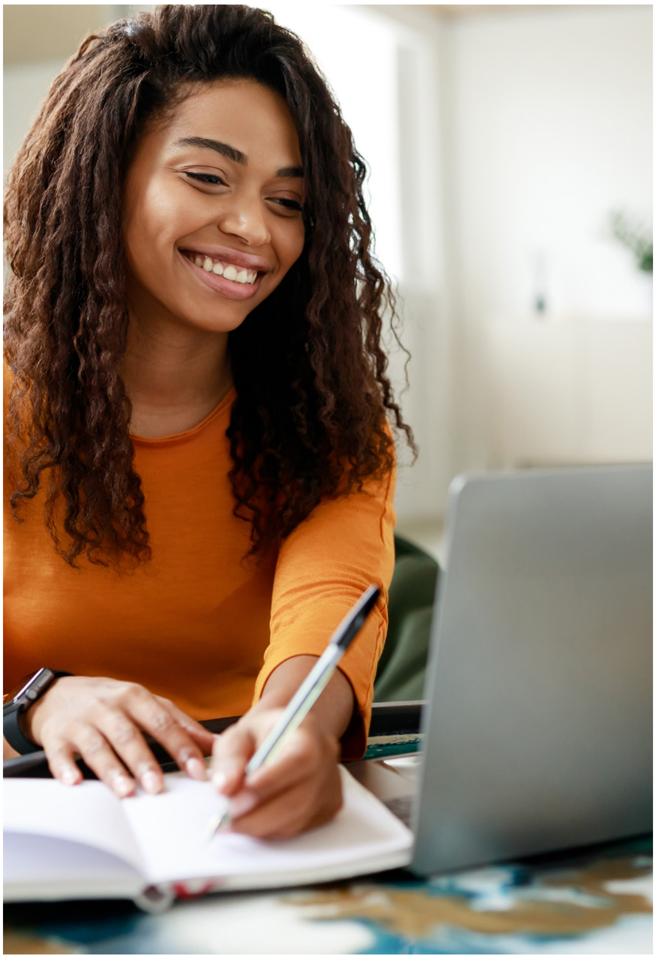

5. REFLECTIONS

GOING HOME CHECKLIST

It is important to take the time to reflect on your experiences and what you have learnt. Why not consider the following questions to help you reflect on your time with the ACT.

- ✓ Have I had an opportunity to discuss any concerns?
- ✓ Do I have any questions or concerns I need to discuss before I go home?
- ✓ Have I had a productive day/placement?
- ✓ Have I enjoyed the placement?
- ✓ Would I recommend the alcohol care team as a placement?
- ✓ What are your top 3 takeaways from your placement?

1.	
2.	
3.	

APPENDIX 1: SEVERITY OF ALCOHOL USE DISORDER. (SADQ)

(Stockwell, Hodgson et al. 1979; Stockwell, Murphy et al. 1983)

SEVERITY OF ALCOHOL DEPENDENCE QUESTIONNAIRE (SADQ)

Please recall a typical period of heavy drinking in the last 6 months. When was this? __ / __ / __

Please select a number (either **0**, **1**, **2**, or **3**) to show how often each of the following statements applied to you during this time.

QUESTIONS	ALMOST NEVER	SOME TIMES	OFTEN	NEARLY ALWAYS
I woke up feeling sweaty.	0	1	2	3
My hands shook first thing in the morning.	0	1	2	3
My whole body shook violently first thing in the morning.	0	1	2	3
I woke up absolutely drenched in sweat.	0	1	2	3
I dreaded waking up in the morning.	0	1	2	3
I was frightened of meeting people first thing in the morning.	0	1	2	3
I felt at the edge of despair when I awoke.	0	1	2	3
I felt very frightened when I awoke.	0	1	2	3
I liked to have a morning drink.	0	1	2	3
I always gulped my first few morning drinks down as quickly as possible.	0	1	2	3
I drank in the morning to get rid of the shakes.	0	1	2	3
I had a very strong craving for drink when I awoke.	0	1	2	3
I drank more than 1/4 bottle of spirits a day (or 4 pints of beer/1 bottles of wine).	0	1	2	3
I drank more than 1/2 bottle of spirits a day (or 8 pints of beer/2 bottles of wine)	0	1	2	3
I drank more than 1 bottle of spirits a day (or 15 pints of beer/3 bottles of wine).	0	1	2	3
I drank more than 2 bottles of spirits a day (or 30 pints of beer/4 bottles of wine)	0	1	2	3

Imagine the following situation: (a) You have been **completely** off drink for a **few weeks**.
(b) You then drink **very heavily for two days**.

How would you feel **the morning after** those two days of heavy drinking?

SYMPTOMS	NO	SLIGHT	MODERATE	A LOT
I would start to sweat.	0	1	2	3
My hands would shake.	0	1	2	3
My body would shake.	0	1	2	3
I would be craving for a drink	0	1	2	3

TOTAL SADQ SCORE = __________

Interpretation: Severity of Alcohol Dependence Score:

Score:
0 - 7 Non-dependent
8 - 15 Mild dependence
16 - 30 Moderate dependence
31 – 60 Severe dependence

Significance

8-15 Mild dependence – target goal can be safe drinking, with tightly monitored alcohol consumption.

16-30 Moderate dependence – a period of abstinence will probably be necessary. 80% may be able to control their drinking if it is very carefully reintroduced, monitored closely, and if family support is good. 20% will not be able to achieve this. If relapse occurs, next time aim for abstinence.

31-60 Severe dependence – the target goal is abstinence, although it appears that up to 20% may be able at some point to resume some form of controlled drinking but usually leading to troubled drinking behaviour. These patients will usually require several treatment episodes, good support and energetic treatment of co-morbid physical and psychological problems.

APPENDIX 2:
ADAPTED CLINICAL ALCOHOL WITHDRAWAL ASSESSMENT SCALE (CIWA-AD) (SADQ)

(Reoux, J. P., & Oreskovich, M. R. (2006). A Comparison of Two Versions of the Clinical Institute)

		Score								
Temperature	**37.0-37.5°C**<br>**37.6-38.0°C**<br>**Greater than 38.0°C**	**0**<br>**1**<br>**2**								
Pulse	90-95<br>96-100<br>101-105<br>106-110<br>111-120<br>Greater than 120	1<br>2<br>3<br>4<br>5<br>6								
Respiratory Rate	20-24<br>Greater than 24	1<br>2								
Tremor<br>Arms extended<br>fingers spread	No tremor<br>Not visible—can be felt fingertip to fingertip<br>Moderate with arms extended<br>Severe even with arms not extended	0<br>1<br>4<br>6								
Sweating<br>Observation	No sweat visible<br>Barely perceptible, palms moist<br>Beads of sweat visible<br>Drenching sweats	0<br>1<br>4<br>6								
Clouding of<br>sensorium<br>What day is this?<br>What is this place?	Orientated<br>Disorientated for date by no more than two days<br>Disorientated for date by more than two days<br>Disorientated for place /person	0<br>2<br>3<br>4								
Quality of Contact	In contact with examiner<br>Seems in contact, but is oblivious to environment<br>Periodically becomes detached<br>6Makes no contact with examiner	0<br>2<br>4<br>6								

		Score								
**Agitation observation**	**Normal activity**<br>**Somewhat more than normal activity**<br>**Moderately fidgety and restless**<br>**Pacing, or thrashing about constantly**	**0**<br>**1**<br>**4**<br>**6**								
**Visual Disturbances**	Not present<br>Mild sensitivity (bothered by the lights)<br>Intermittent visual hallucinations (occasionally sees things you cannot)<br>Continuous visual hallucinations (seeing things constantly)	0<br>2<br>4<br>6								
**Thought disturbances (flight of ideas, paranoid ideas)**	No disturbance<br>Does not have much control over nature of own thoughts<br>Constantly troubled by unpleasant thoughts<br>Thoughts come too rapidly and in a disconnected fashion	0<br>2<br>4<br>6								
**NOTE CHANGE IN SCORE Score hourly for first 4 hours.**		Total Score								

APPENDIX 3: GLASGOW MODIFIED ALCOHOL WITHDRAWAL SCALE (GMAWS)

**Tremor**<br>0) No tremor<br>1) On movement<br>2) At rest	
**Sweating**<br>0) No sweat visible<br>1) Moist<br>2) Drenching sweats	
**Hallucination**<br>0) Not present<br>1) Dissuadable<br>2) Not Dissuadable	
**Orientation**<br>0) Orientated<br>1) Vague, detached<br>2) Disorientated, no contact	
**Agitation**<br>0) Calm<br>1) Anxious<br>2) Panicky	
**Score**	
**Treatment**	

Score: (Do not use scoring tool if patient intoxicated, must be at least 8 hours since last drink)

0: Repeat Score in 2 hours (discontinue after scoring on 4 consecutive occasions, except if less than 48hrs after last drink)

1-3: Give 10mg Diazepam: Repeat Score in 2 hours

4-8: Give 20mg Diazepam: Repeat Score in 1 hour

9-10: Give 20mg Diazepam: Repeat Score in 1 hour, discuss with medical staff

A. McPherson, G. Benson, E.H. Forrest, Appraisal of the Glasgow assessment and management of alcohol guideline: a comprehensive alcohol management protocol for use in general hospitals, QJM: An International Journal of Medicine, Volume 105, Issue 7, July 2012, Pages 649-656

APPENDIX 4: MONTREAL COGNITIVE ASSESSMENT (MOCA)

MONTREAL COGNITIVE ASSESSMENT (MOCA)
Version 7.1 Original Version

NAME :
Education : Date of birth :
Sex : DATE :

VISUOSPATIAL / EXECUTIVE

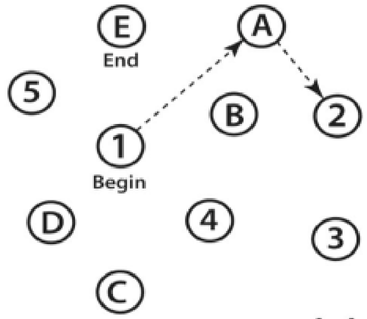

[ ]

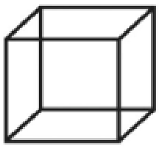

Copy cube [ ]

Draw CLOCK (Ten past eleven)
(3 points)

[ ] Contour [ ] Numbers [ ] Hands

POINTS: ___/5

NAMING

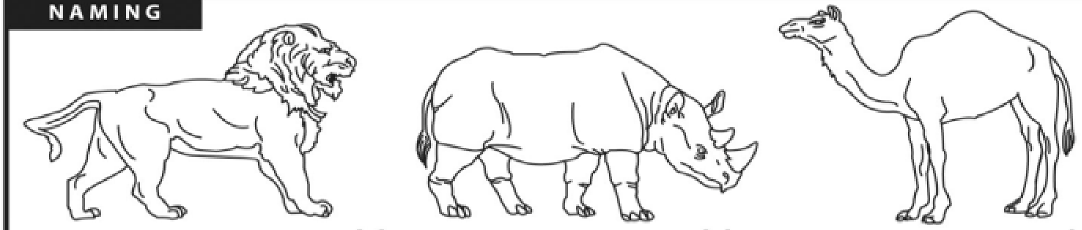

[ ] [ ] [ ] ___/3

MEMORY

Read list of words, subject must repeat them. Do 2 trials, even if 1st trial is successful. Do a recall after 5 minutes.

	FACE	VELVET	CHURCH	DAISY	RED
1st trial					
2nd trial					

No points

ATTENTION

Read list of digits (1 digit/ sec.). Subject has to repeat them in the forward order [ ] 2 1 8 5 4
Subject has to repeat them in the backward order [ ] 7 4 2 ___/2

Read list of letters. The subject must tap with his hand at each letter A. No points if ≥ 2 errors
[ ] F B A C M N A A J K L B A F A K D E A A A J A M O F A A B ___/1

Serial 7 subtraction starting at 100 [ ] 93 [ ] 86 [ ] 79 [ ] 72 [ ] 65
4 or 5 correct subtractions: **3 pts**, 2 or 3 correct: **2 pts**, 1 correct: **1 pt**, 0 correct: **0 pt** ___/3

LANGUAGE

Repeat : I only know that John is the one to help today. [ ]
The cat always hid under the couch when dogs were in the room. [ ] ___/2

Fluency / Name maximum number of words in one minute that begin with the letter F [ ] ___ (N ≥ 11 words) ___/1

ABSTRACTION

Similarity between e.g. banana - orange = fruit [ ] train – bicycle [ ] watch - ruler ___/2

DELAYED RECALL

Has to recall words WITH NO CUE	FACE [ ]	VELVET [ ]	CHURCH [ ]	DAISY [ ]	RED [ ]	Points for UNCUED recall only
Optional: Category cue						
Multiple choice cue						

___/5

ORIENTATION

[ ] Date [ ] Month [ ] Year [ ] Day [ ] Place [ ] City ___/6

© Z.Nasreddine MD www.mocatest.org Normal ≥26 / 30 TOTAL ___/30
Add 1 point if ≤ 12 yr edu

Administered by: ___

Nasreddine, Z.S., Phillips, N.A., Bédirian, V., Charbonneau, S., Whitehead, V., Collin, I., Cummings, J.L. and Chertkow, H., 2005. The Montreal Cognitive Assessment, MoCA: a brief screening tool for mild cognitive impairment. Journal of the American Geriatrics Society, 53(4), pp.695-699.

APPENDIX 5: PATIENT HEALTH QUESTIONNAIRE AND GENERALIZED ANXIETY DISORDER QUESTIONNAIRE

1. Patient Health Questionnaire (PHQ-2 and PHQ-9)

Purpose

The Patient Health Questionnaire (PHQ) is designed to facilitate the recognition and diagnosis of the most common mental disorders in primary care patients. For patients with a depressive disorder, a PHQ Depression Severity Index score can be calculated and repeated over time to monitor change.

Who Should Take the PHQ The PHQ should be used with all new patients,

Making a Diagnosis Since the questionnaire relies on patient self-report, definitive diagnoses must be verified by the clinician, taking into account how well the patient understood the questions in the questionnaire, as well as other relevant information from the patient, his or her family or other sources.

Interpreting the PHQ To facilitate interpretation of patient responses, all clinically significant responses are found in the column farthest to the right (the only exception is for suicidal ideation when diagnosing a depressive syndrome). At the bottom of each page, beginning with “FOR OFFICE CODING”, in small type, are criteria for diagnostic judgments for summarizing the responses on that page. The names of the categories are abbreviated, e.g., Major Depressive Syndrome is Maj Dep Syn. Additional Clinical Considerations After making a provisional diagnosis with the PHQ, there are additional clinical considerations that may affect decisions about management and treatment.

- Have current symptoms been triggered by psychosocial stressor(s)?
- What is the duration of the current disturbance and has the patient received any treatment for it?
- To what extent are the patient’s symptoms impairing his or her usual work and activities?
- Is there a history of similar episodes, and were they treated?
- Is there a family history of similar conditions?

Validation

The PHQ-9 is the depression module, which scores each of the nine DSM-IV criteria as “0” (not at all) to “3” (nearly every day).

It has been validated for use in primary care.

It is not a screening tool for depression, but it is used to monitor the severity of depression and response to treatment. However, it can be used to make a tentative diagnosis of depression in at-risk populations – e.g., those with AUD.

When screening for depression the Patient Health Questionnaire (PHQ-2) can be used first (it has a 97% sensitivity and a 67% specificity). If this is positive, the PHQ-9 can then be used, which has 61% sensitivity and 94% specificity in adults.

Over the last 2 weeks, how often have you been bothered by the following problems? (Use “✔” to indicate your answer)

	Not at all	Several days	More than half the days	Nearly every day
Feeling nervous, anxious, or on edge	0	1	2	3
Not being able to stop or control worrying	0	1	2	3

Comprehensive validation of the PHQ-9 in a psychiatric sample.

- A cut-off of ≥13 yielded good sensitivity and specificity.
- PHQ-9 demonstrated good convergent and discriminant validity.
- PHQ-9 was sensitive to changes following acute treatment.

Beard, C., Hsu, K.J., Rifkin, L.S., Busch, A.B. and Björgvinsson, T., 2016. Validation of the PHQ-9 in a psychiatric sample. Journal of Affective Disorders, 193, pp.267-273.

PHQ-9 score ≥10 had a sensitivity of 88% and a specificity of 88% for major depression. PHQ-9 scores of 5, 10, 15, and 20 represented mild, moderate, moderately severe, and severe depression, respectively.

Kroenke, K., Spitzer, R.L. and Williams, J.B.W. (2001), The PHQ-9. Journal of General Internal Medicine, 16: 606-613. https://doi.org/10.1046/j.1525-1497.2001.016009606.x

**Over the last 2 weeks, how often have you been bothered by any of the following problems?<br><br>(Use "✔" to indicate your answer"**	**Not at all**	**Several days**	**More than half the days**	**Nearly every day**
1. Little interest or pleasure in doing things.	0	1	2	3
2. Feeling down, depressed, or hopeless.	0	1	2	3
3. Trouble falling or staying asleep, or sleeping too much	0	1	2	3
4. Feeling tired or having little energy	0	1	2	3
5. Poor appetite or overeating	0	1	2	3
6. Feeling bad about yourself — or that you are a failure or have let yourself or your family	0	1	2	3
7. Trouble concentrating on things, such as reading the newspaper or watching television	0	1	2	3
8. Moving or speaking so slowly that other people could have noticed? Or the opposite — being so fidgety or restless that you have been moving .around a lot more than usual.	0	1	2	3
9. Thoughts that you would be better off dead or of hurting yourself in some way	0	1	2	3
**Column totals** ____ + ____ + _____ + ____				

Kroenke, K., Spitzer, R.L. and Williams, J.B.W. (2001), The PHQ-9. Journal of General Internal Medicine, 16: 606-613. https://doi.org/10.1046/j.1525-1497.2001.016009606.x

2. Generalized Anxiety Disorder Questionnaire (GAD-7)

The GAD-7 is a valid and efficient tool for screening for GAD and assessing its severity in clinical practice.

**Over the <u>last 2 weeks</u>, how often have you been bothered by any of the following problems?** <br>**(Use "✔" to indicate your answer")**	**Not at all**	**Several days**	**More than half the days**	**Nearly every day**
1. Feeling nervous, anxious or on edge	0	1	2	3
2. Not being able to stop or control worrying	0	1	2	3
3. Worrying too much about different things	0	1	2	3
4. Trouble relaxing	0	1	2	3
5. Being so restless that it is hard to sit still	0	1	2	3
6. Becoming easily annoyed or irritable	0	1	2	3
7. Feeling afraid as if something awful might happen	0	1	2	3
**Column totals** ___ + ___ + ____ + ___				

If you checked off <u>any</u> problems, how <u>difficult</u> have these problems made it for you to do your work, take care of things at home, or get along with other people?

**Not difficult at all**	**Somewhat difficult**	**Very difficult**	**Extremely difficult**

Spitzer, R.L., Kroenke, K., Williams, J.B. and Löwe, B., 2006. A brief measure for assessing generalized anxiety disorder: the GAD-7. Archives of internal medicine, 166(10), pp.1092-1097.

REFERENCES

1	The Nursing and Midwifery Council (NMC) Standards for Pre-registration Nursing Programmes (NMC 2018a)
2	Moriarty KJ , Cassidy P , Dalton D , et al<br>Alcohol-Related disease: meeting the challenge of improved quality of care and better use of resources. A Joint Position Paper on behalf of the British Society of Gastroenterology, Alcohol Health Alliance UK & British Association for Study of the Liver, 2010
3	Alcohol care teams: reducing acute hospital admissions and improving quality of care. 2016. NICE quality and productivity: proven case study. provided by the British Society of Gastroenterology and Bolton NHS Foundation trust. Available: http://www.nice.org.uk/localPractice/collection
4	Owens, L., Kolamunnage-Dona, R., Owens, A., Perkins, E., Butcher, G., Wilson, K., . . Pirmohamed, M. (2016). A Randomized Controlled Trial of Extended Brief Intervention for Alcohol-Dependent Patients in an Acute Hospital Setting. Alcohol and Alcoholism, 51(05), 584-592. doi:10.1093/alcalc/agw023
5	Alcohol-use disorders: prevention<br>Public health guideline [PH24] 2010. https://www.nice.org.uk/guidance/ph24/chapter/Recommendations#recommendation-9-screening-adults
6	Saunders, J.B., Aasland, O.G., Babor, T.F., De la Fuente, J.R. and Grant, M., 1993. Development of the alcohol use disorders identification test (AUDIT): WHO collaborative project on early detection of persons with harmful alcohol consumption-II. Addiction, 88(6), pp.791-804)
7	Conigrave KM, Hall WD, Saunders JB. The AUDIT questionnaire: choosing a cut-off score. Alcohol Use Disorder Identification Test. Addiction. 1995 Oct;90(10):1349-56. doi: 10.1046/j.1360-0443.1995.901013496.x. PMID:
8	Aertgeerts, B., Buntinx, F., Ansoms, S. and Fevery, J., 2002. Questionnaires are better than laboratory tests to screen for current alcohol abuse or dependence in a male inpatient population. Acta Clinica Belgica, 57(5), pp.241-249.
9	Bush, K., Kivlahan, D.R., McDonell, M.B., Fihn, S.D., Bradley, K.A. and Ambulatory Care Quality Improvement Project (ACQUIP, 1998. The AUDIT alcohol consumption questions (AUDIT-C): an effective brief screening test for problem drinking. Archives of internal medicine, 158(16), pp.1789-1795.
10	Stockwell, T., Hodgson, R., Edwards, G., Taylor, C. and Rankin, H., 1979. The development of a questionnaire to measure severity of alcohol dependence. British journal of addiction.
11	SHAW, J.M., KOLESAR, G.S., SELLERS, E.M., KAPLAN, H.L. and Sandor, P.A.U.L., 1981. Development of optimal treatment tactics for alcohol withdrawal. I. Assessment and effectiveness of supportive care. Journal of Clinical Psychopharmacology, 1(6), pp.382-389.
12	Reoux, J.P. and Oreskovich, M.R., 2006. A comparison of two versions of the clinical institute withdrawal assessment for alcohol: The CIWA–Ar and CIWA-AD. American Journal on Addictions, 15(1), pp.85-93.
13	McPherson, A., Benson, G. and Forrest, E.H., 2012. Appraisal of the Glasgow assessment and management of alcohol guideline: a comprehensive alcohol management protocol for use in general hospitals. QJM: An International Journal of Medicine, 105(7), pp.649-656.

14	Alcohol-use disorders: diagnosis and management of physical complications Clinical guideline [CG100] 2010 https://www.nice.org.uk/guidance/cg100
15	Foncea, C.G., Moga, T.V., Sporea, I., Popa, A., Sirli, R., Bende, F., Popescu, A. and Fofiu, R., 2022. The role of elastography for liver fibrosis screening in alcoholic liver disease. Medical Ultrasonography, 24(4), pp.406-413.
16	Owens, L., Thompson, A., Siju, C., & Richardson, P. (2018). A six month follow-up study to determine the clinical utility and patient acceptability of fibroelastography in detection and treatment of alcohol-related liver disease. Journal of Hepatology, 68, S365. doi:10.1016/S0168-8278(18)30956-5
17	Owens, L., Thompson, A., Patterson, K. and Richardson, P., 2019. Alcohol-related brain injury: Improving identification and management in an acute care setting. Future Healthcare Journal, 6(Suppl 1), p.84.
18	Nasreddine, Z.S., Phillips, N.A., Bédirian, V., Charbonneau, S., Whitehead, V., Collin, I., Cummings, J.L. and Chertkow, H., 2005. The Montreal Cognitive Assessment, MoCA: a brief screening tool for mild cognitive impairment. Journal of the American Geriatrics Society, 53(4), pp.695-699.
19	Spitzer RL, Kroenke K, Williams JB, et al. A brief measure for assessing generalized anxiety disorder: the GAD-7. Arch Intern Med. 2006 May 22 166(10):1092-7)
20	Kaner, E.F., Dickinson, H.O., Beyer, F., Pienaar, E., Schlesinger, C., Campbell, F., Saunders, J.B., Burnand, B. and Heather, N., 2009. The effectiveness of brief alcohol interventions in primary care settings: a systematic review. Drug and alcohol review, 28(3), pp.301-323.
21	Silagy, C., Lancaster, T., Stead, L.F., Mant, D. and Fowler, G., 2004. Nicotine replacement therapy for smoking cessation. Cochrane database of systematic reviews, (3).
22	Cobain, K., Owens, L., Kolamunnage-Dona, R., Fitzgerald, R., Gilmore, I. and Pirmohamed, M., 2011. Brief interventions in dependent drinkers: A comparative prospective analysis in two hospitals. Alcohol and Alcoholism, 46(4), pp.434-440.
23	Owens, L., Kolamunnage-Dona, R., Owens, A., Perkins, E., Butcher, G., Wilson, K., . . . Pirmohamed, M. (2016). A Randomized Controlled Trial of Extended Brief Intervention for Alcohol-Dependent Patients in an Acute Hospital Setting. Alcohol and Alcoholism, 51(05), 584-592. doi:10.1093/alcalc/agw023
24	Bien, T.H., Miller, W.R. and Tonigan, J.S., 1993. Brief interventions for alcohol problems: a review. Addiction, 88(3), pp.315-336.

USEFUL LINKS TO ORGANISATIONS

Alcohol Care Teams (ACT)	https://www.longtermplan.nhs.uk/wp-content/uploads/2019/11/ACT-what-are-we-proposing-and-why-011119.pdf
Alcohol Change UK	https://alcoholchange.org.uk/
British Society of Gastroenterology (BSG)	https://www.bsg.org.uk/clinical-resource
British Association for the Study of Liver disease (BASL)	https://www.basl.org.uk/
British Liver Trust	https://britishlivertrust.org.uk/
CHAMPS Public Health Collaborative	https://champspublichealth.com/
European Association for the Study of Liver disease (EASL)	https://easl.eu/
Institute of Alcohol Studies	https://www.ias.org.uk/
Liverpool CCG Alcohol Data	https://opendata.ljmu.ac.uk/id/eprint/140/
Lower My Drinking app	https://lowermydrinking-nhs.org.uk/
NDTMS	https://www.ndtms.net/resources/public/Parental%20substance%20misuse/North%20West/NW_Liverpool_2019-20_Parental_substance_misuse_data_pack.html
NHS<br>Long Term Plan	https://www.england.nhs.uk/ourwork/prevention/alcohol-dependency-programme
National Institute for Health and Care Excellence (NICE)	https://www.nice.org.uk/guidance/cg100
National Institute of Health (NIH) National Library of Medicine	https://www.ncbi.nlm.nih.gov/pmc/articles/PMC10099257/
Royal College of Psychiatry (RCPsych)<br>ACTION	https://www.rcpsych.ac.uk/improving-care/ccqi/quality-networks-accreditation/alcohol-care-team-innovation-optimisation-network

NOTES

Champs
Public Health
Collaborative

Champs Public Health Collaborative
PO Box 290
Brighton Street
Wallasey
CH27 9FQ

T: 0151 666 5123
E: champscommunications@wirral.gov.uk

ChampsPublicHealth Collaborative

@ChampsPHC

ChampsPublicHealth Collaborative

@ChampsPHC

@ChampsPHC
