# Supplementary material for: ACTIVE involvement in alcohol care: a community case study in coproduction
Source: Front Public Health. 2026 Jun 18;14:1816664. doi: 10.3389/fpubh.2026.1816664 (PMC13323507; doi:10.3389/fpubh.2026.1816664)

#
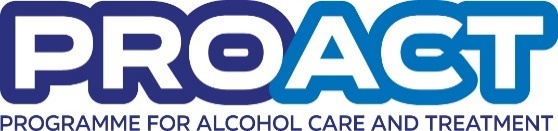


# ACTIVE PPI group – Values and Principles

**A**lcohol **C**are **T**eam **I**n**V**olvement and **E**ngagement group-PPI (patient and public involvement).

## Mission

The group aims to make the most of our experiences of alcohol and mental health care and support, with a view to influencing positive change in relevant services.

We will speak our Truth into services and aim to act as a standing resource for the region’s [Reduction of Harm from Alcohol programme.](https://champspublichealth.com/reduction-of-harm-through-alcohol/)

## Language

We will keep a watchful eye on the language we use and pay attention to how people wish to be referred to. We respect everyone’s personal perspective and point of view.

We ask partners to respect that we are PEOPLE first, not simply patients, service users, clients or carers.

## Membership of the group

By completing and returning our consent form, individuals express their commitment to being part of the group and attending regular meetings.

It is everyone’s responsibility to create a safe, supportive space for people to share personal experiences and ideas for improvement. This requires listening to others and respecting everyone’s right to an opinion.

Members are not expected to share every detail of their lives, only as much as they feel happy to do.

ACTIVE members are encouraged to build a supportive environment in which we can trust one another.

Members are not expected to attend every meeting.

Members can let the organiser know if they are unable to attend a meeting or need some time out from the group.

If people are absent for 3 meetings or more, facilitators will contact them to determine if they still wish to remain involved.

Facilitators (organisers) from the University of Lancashire Comensus group will organise and invite members to the group.

Facilitators will respect members’ rights to privacy and confidentiality. Personal details will not be shared with other professionals connected to the PROACT network without express permission.

Group members and facilitators agree that they should not be under the influence of alcohol or other substances when attending meetings. However, we will not exclude people who are still seeking help from services.

People are free to raise their concerns about other members within the group; the facilitators and other professionals will deal with any issues that arise on an individual basis.

## Flexibility

Meetings will normally take place monthly.

The group will be flexible in terms of venues for meetings, online meetings and frequency of meetings. Members will be consulted regarding the accessibility of venues.

Where necessary, hybrid meetings will be offered as well as online meetings during periods of bad weather or concerns regarding a future pandemic. Face to face meetings will be our preferred option.

## No Hierarchy

Members are able to disagree with others, however we will respect other members’ right to be controversial or have their own opinion. Tasks will be allocated to individuals according to members’ interests and strengths.

# Notes will be taken by the facilitator of the group or another member if they wish to volunteer. Other administration will be undertaken by the facilitator.

## Duration of the group

## The ACTIVE PPI group is time-limited for now, but the long-term aim is for ACTIVE group to be a ‘standing committee’ or consultative body for the PROACT Network and CHAMPS Public Health team as they bid to improve and re-design public health services.

# Guide to Abbreviations and acronyms

**ACT** Alcohol Care team

**ACTIVE** Alcohol Care Team Involvement and Engagement Group

**CHAMPS** Cheshire and Merseyside Public Health Services

**COMENSUS** Community Engagement and Service User Support group at University of Central Lancashire.

**EXAMH** Early identification of Co-occurring Alcohol use and Mental health disorder

**PhAST** Physical activity for people involved in recovery

**PPI** Patient and Public Involvement

**PROACT** Program for Alcohol Care and Treatment


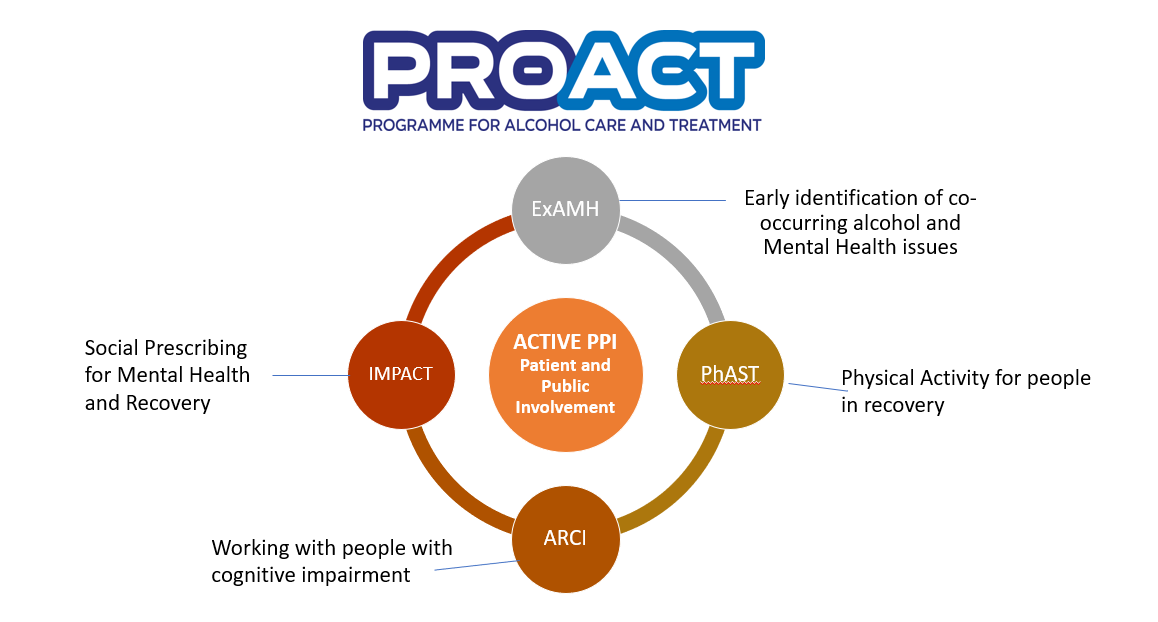

Supplement: Supplementary file 3 [file Data_Sheet_3.docx]
