# Supplementary material for: ACTIVE involvement in alcohol care: a community case study in coproduction
Source: Front Public Health. 2026 Jun 18;14:1816664. doi: 10.3389/fpubh.2026.1816664 (PMC13323507; doi:10.3389/fpubh.2026.1816664)
Supplement: Supplementary file 4 [file Data_Sheet_4.docx]

**Terms of this Agreement**

| Details of the parties | | |
| --- | --- | --- |
|  | **Client** | **Contractor** |
| Name | Cheshire and Merseyside Directors of Public Health | University of Lancashire |
| Address | Cheshire Lines Building, Canning Street, Birkenhead, Wirral, Merseyside, CH41 1ND | University of Lancashire Preston PR1 2HE |
| Contact person: | Redacted | Redacted |
| Contact person’s telephone number(s) | Redacted | Redacted |
| Contact person’s email: | Redacted | Redacted |
| Reference to the contact person of a party includes any replacement contact person as communicated to the other party from time to time | | |

| Background | |
| --- | --- |
| Background to this Agreement: | - The aims of the Client are (among other things) are to enable local health and care professionals to work together collaboratively for the benefit of the people of Cheshire and Merseyside. - Patient and Public Involvement (PPI) support is required for the Programme for Alcohol Care and Treatment (PROACT) and it’s linked workstreams within the Cheshire and Merseyside ‘Reduction of Harm from Alcohol’ programme. - The Client requires the services of a suitably qualified PPI provider to further its aims. - The Contractor has certain skills and experience which can be made available to the Client to further its aims. |

| Duration of Services | |
| --- | --- |
| Commencement date of the Services | 1^st^ April 2025 |
| Expiry date of the Services | 31^st^ March 2026 |

| The Services | |
| --- | --- |
| The Services which the Contractor must provide the Client: | See the specification in Appendix 2. |
| Whether Services are to be provided for the benefit of anyone else in addition to the Client: | The delivery of PPI support will benefit patients under the care of alcohol services across Cheshire and Merseyside. |

| Working hours etc. | |
| --- | --- |
| Days on which the Contractor is expected to work | - Such hours shall be provided on such days of the week as are reasonably required by the Client and as agreed with the Contractor. |
| Working hours | - Hours as agreed with the Client and the Contractor. |
| Approvals, notifications and other arrangements if the Contractor is to be absent (e.g. holidays, illness etc) | - The Contractor shall communicate any absence which prevents them from carrying out the required number of hours in a week (e.g., due to holidays, illness etc). |

| Location, facilities | |
| --- | --- |
| Location where the Services are to be carried out: | - Services will be carried out at the Client’s premises or at other venues as determined by the needs of the work being undertaken. |
| Provision of facilities: | - Provision of facilities and support required to provide the Services (e.g. desk, computer, access to network) will be arranged by the Contractor |

| Fees | |
| --- | --- |
| Fees payable by the Client to the Contractor in consideration for the Services (including VAT and similar taxes): | £12,075.94 (inclusive of VAT) |
| How the Fees change over time (e.g. regular increases): | No changes to Fee rates are expected during the term of the agreement. |
| Consequences on the Fees if the Contractor cannot provide the required services | If no services are provided as per schedule in Appendix 2, then no Fees shall be payable. |
| The Fees shall be paid without deduction or set-off (unless the Client has a valid and recognised judgment against the Contractor for a fixed sum, in which case that sum may be set off). |  |
| All sums due from the Client to the Contractor which are not paid on the due date shall bear interest from day to day at the annual rate of 4% over the base rate from time to time of the Council’s designated bank. |  |

| Termination | | |
| --- | --- | --- |
| Termination ‘for convenience’: right of a party to terminate this Agreement even if no event of default then applies to the other party: | | |
|  | **Client’s right** | **Contractor’s right** |
| Whether the party may do so | It may do so. | It may do so. |
| Procedure to terminate | By notice to the Contact Person at any time after the date of this Agreement. | By notice to the Contact Person at any time after the date of this Agreement. |
| Notice period (at the end of which the termination becomes effective) | 30 days after the notice is given. | 30 days after the notice is given. |
| Any termination fee payable by the terminating party to the other party | Return of fee paid on pro-rata basis | Nil. |

**Appendix 1 – Rules relating to this Agreement**

| General obligations of the Contractor | |
| --- | --- |
| Standards to which the Contractor must provide the Services: | The highest of the following:  - Standards required by Law. - With reasonable skill, care and diligence. |
| How the Contractor must ensure they conduct themselves in providing the Services | - In a lawful, professional, honest, ethical, punctual, safe, tidy and courteous manner. - Using their best available knowledge, subject to any genuine confidentiality obligations she owes to third parties. |
| Obligations if the Contractor has a conflict of interest in relation to any matter affecting the Client: | The Contractor must declare it in a timely and proper manner on becoming aware of it. |

| Invoicing and payment | |
| --- | --- |
| When the Contractor may invoice the Client (e.g. frequency, dates, occurrence of events etc): | Invoices to be submitted by the Contractor as per the schedule below:   - £3,018.99 on 16^th^ June 2025 - £3,018.99 on 15^th^ September 2025 - £3,018.98 on 15^th^ December 2025 - £3,018.98 on 16^th^ March 2026 |
| Due date for payment by the Client of amounts invoiced | 30 days after the invoice is issued. |
| Required method of payment of invoices and claims for expenses | BACS payment in the relevant nominated bank account of the Contractor. |

| Intellectual Property | |
| --- | --- |
| To whom Intellectual Property arising in the course of the provision of the Services (including any goodwill attached to it) is to belong: | - To the Client or its nominee, and not to the Contractor or anyone else. - Such arising Intellectual Property shall vest in the Client or its nominee instantly as it arises. - The Client shall grant to the Contractor an irrevocable, non-exclusive, royalty free licence to use any Intellectual Property arising from the Contractor’s provision of the Services for its academic, research and publication purposes. - The Contractor will use its reasonable endeavours to ensure the Services are delivered in accordance with accepted scientific and other principles and standards, but makes no representation or warranty that any the Services will lead to any specific result or to the creation of any Intellectual Property and accepts no responsibility for any use which may be made of any Intellectual Property arising from the Services. It is therefore agreed that either party utilising such Intellectual Property is fully responsible and liable for any loss, costs, claims or demands arising from that use. - Any publications arising from the Services shall be decided in accordance with normal academic practice. |

| Exit obligations | |
| --- | --- |
| Obligations of the Client on termination of this Agreement: | To promptly and properly return to the Contractor any property of the Contractor then in the possession or control of Client in connection with the Services. |
| Obligations of the Contractor on termination of this Agreement: | To do the following in a prompt and proper manner:  - To return to the Client any property of the Client then in the possession or control of the Contractor. This includes, without limitation and where relevant, any files or other records, and any keys or security passes or the like. - To remove from all electronic storage devices of the Contractor or the Key Personnel the following: - Software belonging to or licensed by the Client. - Any electronic files relevant to the Services. |

| Confidentiality | |
| --- | --- |
| Type of information covered as Confidential Information of the Client: | - Information relating to the Client’s business activities generally, including without limitation, its business strategies, plans, finances, operations, Personnel, products or services, research activities, customers or clients or unpublished Intellectual Property - It shall be deemed to include information of third parties in relation to which the Client are under a duty of confidentiality, to the extent the Contractor knows or reasonably ought to know of that duty of confidentiality. |
| Exceptions to the obligations in clause 12 | Where any of the following applies from time to time:   - Where the express or clearly implied consent of the Client is given. - Where reasonably necessary in the course of providing the Services, subject to the Contractor complying with reasonable directions of the Client regarding the protection of the confidentiality of the information. - Where compelled by Law (including any relevant Law relating to freedom of information) to disclose the Confidential Information, subject to the Contractor doing the following: - Communicating the compelled required to the Client in a timely manner. - Providing (at the Client’s reasonable cost) the Client with reasonable cooperation (if requested) to enable the Client to challenge the compelled disclosure. |

| Miscellaneous | |  |
| --- | --- | --- |
| How this Agreement is to be validly amended (and no other way shall be valid, including the other conduct of the parties): | - By agreement in writing of the parties. - It must be clear in the written document that it is intended to amend this Agreement. | |

15. **Limitation of Liability**

15.1 Subject to clauses 15.2 and 15.3 below, the entire liability of the Contractor arising under or in connection with Agreement, whether in contract, tort (including negligence), breach of statutory duty or otherwise, is limited to the value of the Fee in the aggregate.

15.2 Subject to clause 15.3 below, the Contractor shall not be liable to the Client for loss of business or revenue, loss of profits, loss of anticipated savings, injury to reputation, loss of goodwill, wasted expenditure or any indirect, special or consequential losses or damages, howsoever arising, under or in connection with this Agreement.

15.3 Nothing in this Agreement shall operate to restrict or exclude either Party’s liability for death or personal injury caused by negligence or any other liability which cannot be restricted or excluded by law.

15.4 The Client undertakes to make no claim in connection with this Agreement or its subject matter against any individual employee, student, agent or appointee of the Contractor (apart from claims based on fraud or wilful misconduct).

**Appendix 2 – Project Background and Services to be Provided**

**Background**

The Cheshire and Merseyside reduction of harm from alcohol programme aims to deliver preventative, early detection, and treatment interventions through integrated system working to reduce alcohol harms.

To build an effective, person-centred programme, it is important to engage with people with lived experience of alcohol use disorder (AUD) in a meaningful way to better understand and address the barriers to accessing support and any associated stigma.

To enable this meaningful engagement, a patient and public involvement (PPI) group consisting of local people with lived experience of AUD, including family and carers, can provide a protected space for open discussion on how improve the support available and ultimately reduce alcohol harms. By working alongside the PPI group, the reduction of harm from alcohol programme team can tailor the programme to better meet the needs of people with AUD.

An effective PPI group requires facilitation by an experienced community engager to foster a safe, consistent and supportive environment. They will help empower the PPI members and encourage peer support. The reduction of harm from alcohol programme will fund this role and provide a budget to cover expenses for the PPI members.

**Requirements**

- Experience of setting up and facilitating groups with seldom heard members of the public with lived experience of co-morbidities.
- The ability to develop strong community links to identify group members from within Cheshire and Merseyside with lived experience.
- The ability to retain group members by offering a supportive and mutually beneficial environment.

**Specification**

ACTIVE PPI Specification

1. Organise and facilitate the PPI group meetings

- Over a one-year period, deliver up to 6 meetings with just the lived experience members and a further 4 meetings where wider partners (e.g. clinicians and commissioners) can also attend.
- Act as the point of contact for the group members.
- Set and distribute the agenda at least one week in advance of the meetings.
- Using the funding provided, book the venue for the face-to-face meetings within Cheshire and Merseyside, being considerate of accessibility requirements (including public transport links and parking availability) and the distance the PPI members would need to travel.
- Arrange refreshments for the meetings.
- Chair the meeting, allowing everyone’s voices to be heard.
- Record and distribute the minutes from the meeting, being sure to review them for accuracy at the following meeting.
- Using the funding provided, reimburse PPI group members’ expenses and pay members for their time.

1. Monitor and review membership of the PPI group and expand as required, considering the needs of the programme, experience and interest of group members, geographical spread, and diversity (as appropriate given patient profile / epidemiology).
2. Attendance and presentations (if requested) at the bi-annual Programme for Alcohol Care and Treatment (PROACT) Clinical Network Events on the work of the PPI group.
3. Provide support (when requested) to the Cheshire and Merseyside Reduction of Harm from Alcohol programme through the PPI group including:

- Co-designing patient facing resources.
- Advising on the potential impacts of pathways and interventions from a patient and family perspective.
- Reviewing evaluations to provide a patient and family perspective.
- Advice on project key performance indicators (KPIs).
- Co-development of the process to support getting feedback from patients/family/friends/carers on ways to improve alcohol treatment services.

1. Support the PPI group to review the feedback received from people accessing alcohol care services and their friends, family or carers. Lead on the monitoring of the feedback and ensure it is reviewed by the PPI group.

- Print and deliver the feedback surveys, collection boxes and feedback posters to Cheshire and Merseyside Trusts.
- Monitor incoming feedback received through the surveys.
- Set the agenda and facilitate the PPI group discussion on the feedback.
- Draft the PPI group’s response to the feedback in collaboration with the PROACT clinical and commissioning leads. This will be in the form of a ‘you said, we did’ poster.
- Work with the PROACT clinical and commissioning leads to refine the feedback process and explore expanding to community alcohol care settings.

1. Form relationships and work collaboratively with other alcohol focused lived experience networks within Cheshire and Merseyside.
